# Supplementary material for: Sparse multitask group Lasso for genome-wide association studies
Source: PLoS Comput Biol. 2025 Sep 12;21(9):e1012734. doi: 10.1371/journal.pcbi.1012734 (PMC12448984; doi:10.1371/journal.pcbi.1012734)
Supplement: S1 Appendix — (PDF) [file pcbi.1012734.s001.pdf]

S1 Appendix. SMuGLasso method details

Population structure Diverse and admixed populations studies offer a unique, yet complex, opportunity. On the one hand, they provide an excellent means to increase the number of samples, unlike homogeneous studies where the number of samples is in most data very restricted. Indeed, genotyping hundreds of thousands of participants from different ancestries to study a phenotype of interest helps to alleviate the curse of dimensionality. On the other hand, such analyses require close attention to the confounder raised by population stratification, that is, when association is detected on the population structure rather than on the phenotype of interest. The presence of population stratification is one of the major problems in association studies as it increases type I error and leads to ambiguous results. This is particularly true when allele frequency differences in cases and controls are due to differences in ancestry rather than association between SNPs and disease. To counteract the issue, several correction methods have been developed, including genomic control, Principal Component Analysis (PCA)-based methods [1–8], and Linear mixed models [9–11]. Each of these approaches offers specific benefits and is designed to mitigate the impact of population structure on study results. However, a critical aspect to consider in these adjustment methods is the potential for overcorrection, particularly concerning causal SNPs in certain populations. Additionally, these techniques may not fully account for the presence of population-specific variants that are associated with the disease under study.

Therefore, we observe an existent need in GWAS field to develop efficient frameworks that profit from the advantages provided by diverse studies. Such frameworks should address the issues posed by population stratification, as well as consider the existence of population-specific causal LD-groups.

Consequently, to handle population structure in our model, we assign genetic subpopulations to specific input tasks. This is achieved by using PCA in conjunction with k-means clustering, which allows us to accurately determine the subpopulation to which each sample belongs.

Linkage disequilibrium groups clustering LD causes genetic variants to be correlated, indicating that nearby alleles are inherited together more often than expected by chance, thereby influencing the identification of disease-associated SNPs. Hence, current approaches in association studies use hierarchical clustering to construct LD-groups, effectively grouping variants based on their correlation patterns. Applying feature selection on LD-groups rather than on single SNPs improves remarkably the stability of the selection. By performing the selection at the LD-groups level, the method reduces the selection options for regularization models, and hence addresses the curse of dimensionality, improving the analysis by focusing on correlated groups of genetic variants. In practice, we have used adjacency-constrained hierarchical clustering algorithm to form the LD-groups assigned to SMuGLasso with adjclust R package [12].

Stability selection We use the stability selection procedure developed by [13] where they propose improving the stability using a subsampling method. In this formulation, variable selection is performed repeatedly on subsamples. The subsampling approach can be used to determine the amount of regularization needed to control the familywise error type I rate. Stability selection is a feature selection based method, it can be combined with several existing methods and aims to improve their performance. The procedure relies on computing the stability path, which represents the probability of a feature to be selected across random subsamples, as a function of the regularization parameter.

We denote by I a random subsample of $\{1, \ldots, n\}$ of size $\lfloor n/2 \rfloor$, we call $\widehat{S}^{\lambda}(I)$ the set of features selected by the selection procedure of interest (for example, Lasso), with a hyperparameter λ, on this subsample of the data. For any feature $j \in \{1, \ldots, p\}$, we call $\widehat{\Pi}_j^{\lambda}$ the probability that feature j is selected on a random subsample of size $\lfloor n/2 \rfloor$ of the data. This probability is determined, given m such random subsamples $I_1, I_2, \ldots, I_m$, as the proportion of those subsamples for which the feature selection procedure selects a feature j:

$$\widehat{\Pi}_j^{\lambda} = \frac{1}{m} \sum_{j=1}^{m} \mathbf{1}_{j \in S^{\lambda}(I_j)}.$$

Finally, given a threshold $\frac{1}{2} < \pi_{\text{cutoff}} \leq 1$ (in this work, we used $\pi_{\text{cutoff}} = 0.75$), the stable set of selected features is determined as:

$$\widehat{S}^{\text{stable}} = \{j : \max_{\lambda \in \Lambda} \widehat{\Pi}_j^{\lambda} \geq \pi_{\text{cutoff}}\}.$$

We detail below the Theorem 1 used to bound the number of expected false selected features:

Theorem 1: Stability selection

Let's assume the set of features with non-zero coefficients by $S = \{j : \beta_j \neq 0\}$, and the set of features with zero coefficients by $N = \{j : \beta_j = 0\}$. Assuming that the distribution of $\left\{1_{\{j \in \hat{S}^{\lambda}\}}, j \in N\right\}$ is exchangeable for all $\lambda \in \mathbb{R}^{+}$. Also, assuming that the original procedure is not worse than the random setting, i.e. for any $\lambda \in \mathbb{R}^{+}$:

$$\frac{\mathbb{E}\left(\left|S \cap \hat{S}^{\lambda}\right|\right)}{\mathbb{E}\left(\left|N \cap \hat{S}^{\lambda}\right|\right)} \geq \frac{|S|}{|N|}.$$

The number of falsely selected variables $V = \left|N \cap \hat{S}^{\text{stable}}\right|$ is then bounded by:

$$\mathbb{E}(V) \leq \frac{1}{2\pi_{\text{cutoff}} - 1} \frac{q_{\lambda}^2}{p},$$

where $q_{\lambda} = \mathbb{E}\left(|S_{\lambda}(I)|\right)$ denotes the expected number of selected variables. The desired calibration is to obtain $\mathbb{E}(V) \leq \alpha$ with α small.

The stability of the selection measurement The method is carried out with the Pearson similarity index [14] and represents an extension of [15]. Let's assume $\mathcal{Z} = \{s_1, \ldots, s_M\}$ is the set of M selected features, where each s_u is a subset of the features. The total number of features is denoted by p and the number of features selected on the uth feature set is given by k_u. A set of selected features s_u can be represented by an indicator vector $z_{u,.} \in \{0,1\}^p$, where $z_{u,j} = 1$ if feature j is selected and 0 otherwise. The Pearson correlation between two feature sets s_u and s_v is presented by the following equation:

$$\phi_{\text{Pearson}}\left(s_u, s_v\right) = \frac{\frac{1}{p} \sum_{j=1}^{p}\left(z_{u,j} - \bar{z}_{u,.}\right)\left(z_{v,j} - \bar{z}_{v,.}\right)}{\sqrt{\frac{1}{p} \sum_{j=1}^{p}\left(z_{u,j} - \bar{z}_{u,.}\right)^2} \sqrt{\frac{1}{p} \sum_{j=1}^{p}\left(z_{v,j} - \bar{z}_{v,.}\right)^2}},$$

where $\forall u \in \{1, \ldots, M\}, \bar{z}_{u,.} = \frac{1}{p} \sum_{j=1}^{p} z_{u,j} = \frac{k_u}{p}$

The stability of the selection based on Pearson correlation can be rewritten as follows:

$$\phi_{\text{Pearson}}\left(\mathbf{s}_u, \mathbf{s}_v\right) = \frac{r_{u,v} - \mathbb{E}_{\nabla}\left[r_{u,v}\right]}{p h_u h_v} = \frac{r_{u,v} - \frac{k_u k_v}{p}}{p h_u h_v},$$

where $\forall u \in \{1, \ldots, M\}, h_u = \sqrt{\frac{k_u}{p}\left(1 - \frac{k_u}{p}\right)}$ and $r_{u,v}$ denotes the number of features in common between the feature sets s_u and s_v. $\mathbb{E}_{\nabla}$ is an adjustment term equal to the expected value of $r_{u,v}$ when the feature selection model selects randomly k_u and k_j features from all features p.

When the number of selected features k is the same for all feature sets, assuming S an index of the variability in the choice of features, the Pearson correlation is established as follows:

$$\hat{\Phi}_{\text{Pearson}}(\mathcal{Z}) = 1 - \frac{S}{S_{\max}} = \frac{\frac{1}{p}\sum_{j=1}^{p} s_j^2}{\frac{k}{p}\left(1 - \frac{k}{p}\right)},$$

where $S_{\max}$ is the maximal value of S when the feature selection model selects k features per feature set. $s_j^2 = \frac{M}{M-1}\hat{q}_j\left(1 - \hat{q}_j\right)$ corresponds to the sample variance of selection of the jth feature. $\hat{q}_j$ corresponds to the observed frequency of the selection of a feature j, as well as the sample mean of the variable $\mathcal{Z}_j$.

References

1. Price AL, et al. Principal components analysis corrects for stratification in genome-wide association studies. Nat Genet. 2006;.

2. Zeggini E, et al. Meta-analysis of genome-wide association data and large-scale replication identifies additional susceptibility loci for type 2 diabetes. Nat Genet. 2008;.

3. Need AC, et al. A Genome-Wide Investigation of SNPs and CNVs in Schizophrenia. PLOS Genetics. 2009;.

4. Yu K, Wang Z, et al. Population Substructure and Control Selection in Genome-Wide Association Studies. PLoS One. 2008;.

5. Peloso GM, Timofeev N, Lunetta KL. Principal-component-based population structure adjustment in the North American Rheumatoid Arthritis Consortium data: impact of single-nucleotide polymorphism set and analysis method. BMC Proc. 2009;.

6. Peloso GM, Lunetta KL. Choice of population structure informative principal components for adjustment in a case-control study. BMC Genetics. 2011;.

7. Novembre J, Stephens M. Interpreting principal component analyses of spatial population genetic variation. Nature genetics. 2008;.

8. Qizhai L, Kai Y. Improved Correction for Population Stratification in Genome-wide Association Studies by Identifying Hidden Population Structures. Genetic Epidemiology. 2008;.

9. Kang HM, Zaitlen NA, et al. Efficient Control of Population Structure in Model Organism Association Mapping. Genetics. 2008;.

10. Price AL, Zaitlen NA, et al. New approaches to population stratification in genome-wide association studies. Nat Rev Genet. 2010;.

11. Lippert C, et al. FaST linear mixed models for genome-wide association studies. Nat Methods. 2011;.

12. Ambroise C, et al. Adjacency-constrained hierarchical clustering of a band similarity matrix with application to genomics. Algorithms Mol Biol. 2019;.

13. Meinshausen N, Bühlmann P. Stability Selection. J R Statist Soc B. 2009;.

14. Nogueira S, Brown G. Measuring the Stability of Feature Selection with Applications to Ensemble Methods. International Workshop on Multiple Classifier Systems. 2015;.

15. Kuncheva LI. A stability index for feature selection. IASTED ICAIA. 2008;.
