# Supplementary material for: Sparse multitask group Lasso for genome-wide association studies
Source: PLoS Comput Biol. 2025 Sep 12;21(9):e1012734. doi: 10.1371/journal.pcbi.1012734 (PMC12448984; doi:10.1371/journal.pcbi.1012734)
Supplement: S2 Appendix — (PDF) [file pcbi.1012734.s002.pdf]

S2 Appendix. DRIVE

Data access The dataset "General Research Use" in DRIVE Breast Cancer OncoArray Genotypes is available from the dbGaP controlled-access portal, under Study Accession phs001265.v1.p1 (`https://www.ncbi.nlm.nih.gov/projects/gap/cgi-bin/study.cgi?study_id=phs001265.v1.p1`). Researchers can gain access the data by applying to the data access committee, see `https://dbgap.ncbi.nlm.nih.gov`.

Ethics approval The dataset was obtained from NIH after ethical review of project #17707, titled "Network-guided multi-locus biomarker discovery", and used under approval of this request (#67806-4).

Acknowledgments OncoArray genotyping and phenotype data harmonization for the Discovery, Biology, and Risk of Inherited Variants in Breast Cancer (DRIVE) breast-cancer case control samples was supported by X01 HG007491 and U19 CA148065 and by Cancer Research UK (C1287/A16563). Genotyping was conducted by the Center for Inherited Disease Research (CIDR), Centre for Cancer Genetic Epidemiology, University of Cambridge, and the National Cancer Institute. The following studies contributed germline DNA from breast cancer cases and controls: the Two Sister Study (2SISTER), Breast Oncology Galicia Network (BREOGAN), Copenhagen GeneralPopulation Study (CGPS), Cancer Prevention Study 2 (CPSII), The European Prospective Investigation into Cancer and Nutrition (EPIC), Melbourne Collaborative Cohort Study (MCCS), Multiethnic Cohort (MEC), NashvilleBreast Health Study (NBHS), Nurses Health Study (NHS), Nurses Health Study 2 (NHS2), Polish Breast CancerStudy (PBCS), Prostate Lung Colorectal and Ovarian Cancer Screening Trial (PLCO), Studies of Epidemiologyand Risk Factors in Cancer Heredity (SEARCH), The Sister Study (SISTER), Swedish Mammographic Cohort (SMC), Women of African Ancestry Breast Cancer Study (WAABCS), Women's Health Initiative (WHI).
