# Supplementary figures and images for: Sparse multitask group Lasso for genome-wide association studies
Source: PLoS Comput Biol. 2025 Sep 12;21(9):e1012734. doi: 10.1371/journal.pcbi.1012734 (PMC12448984; doi:10.1371/journal.pcbi.1012734)

# Principal Component Analysis

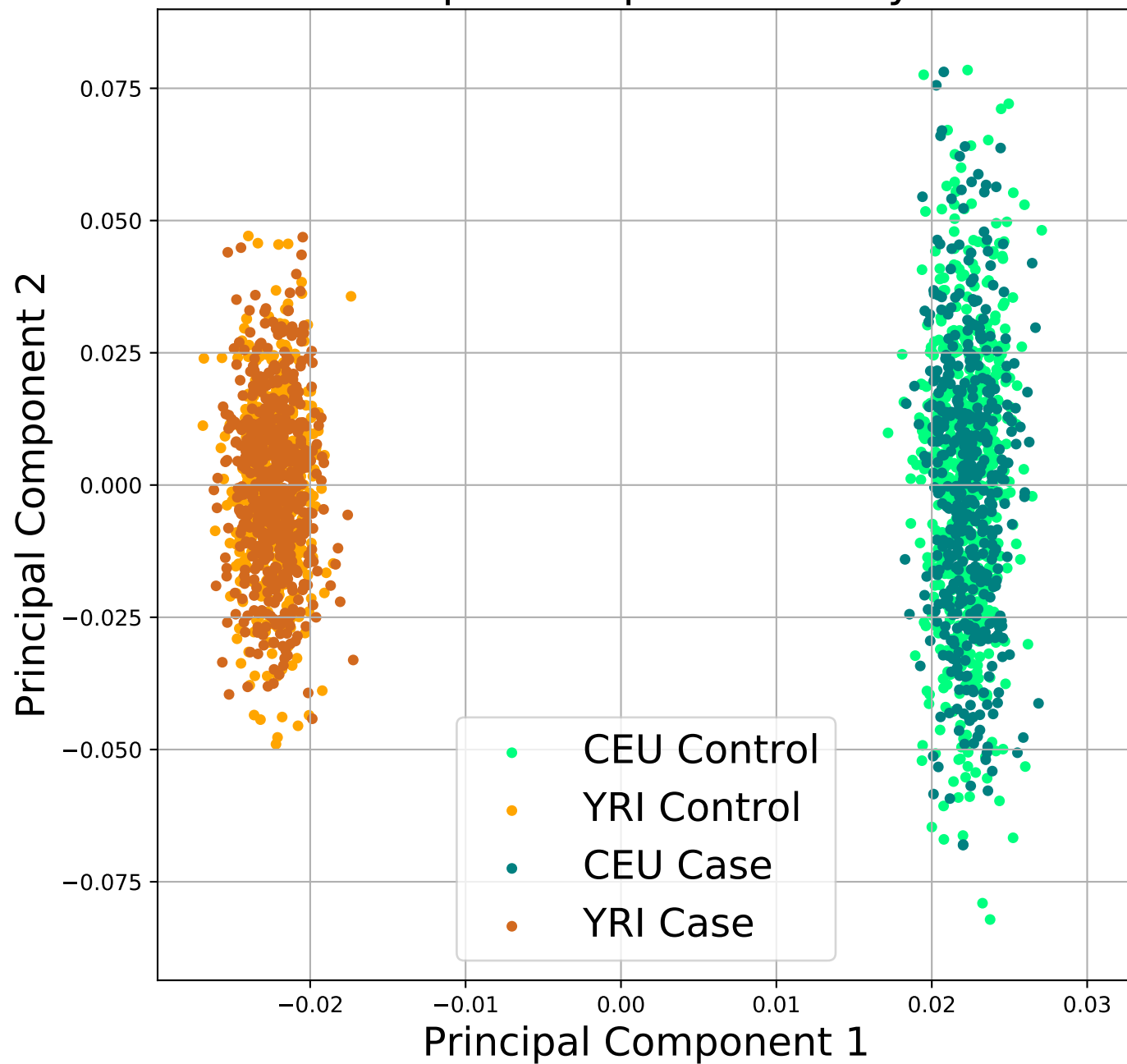

Supplement: S1 Fig — (PDF) [file pcbi.1012734.s003.pdf]

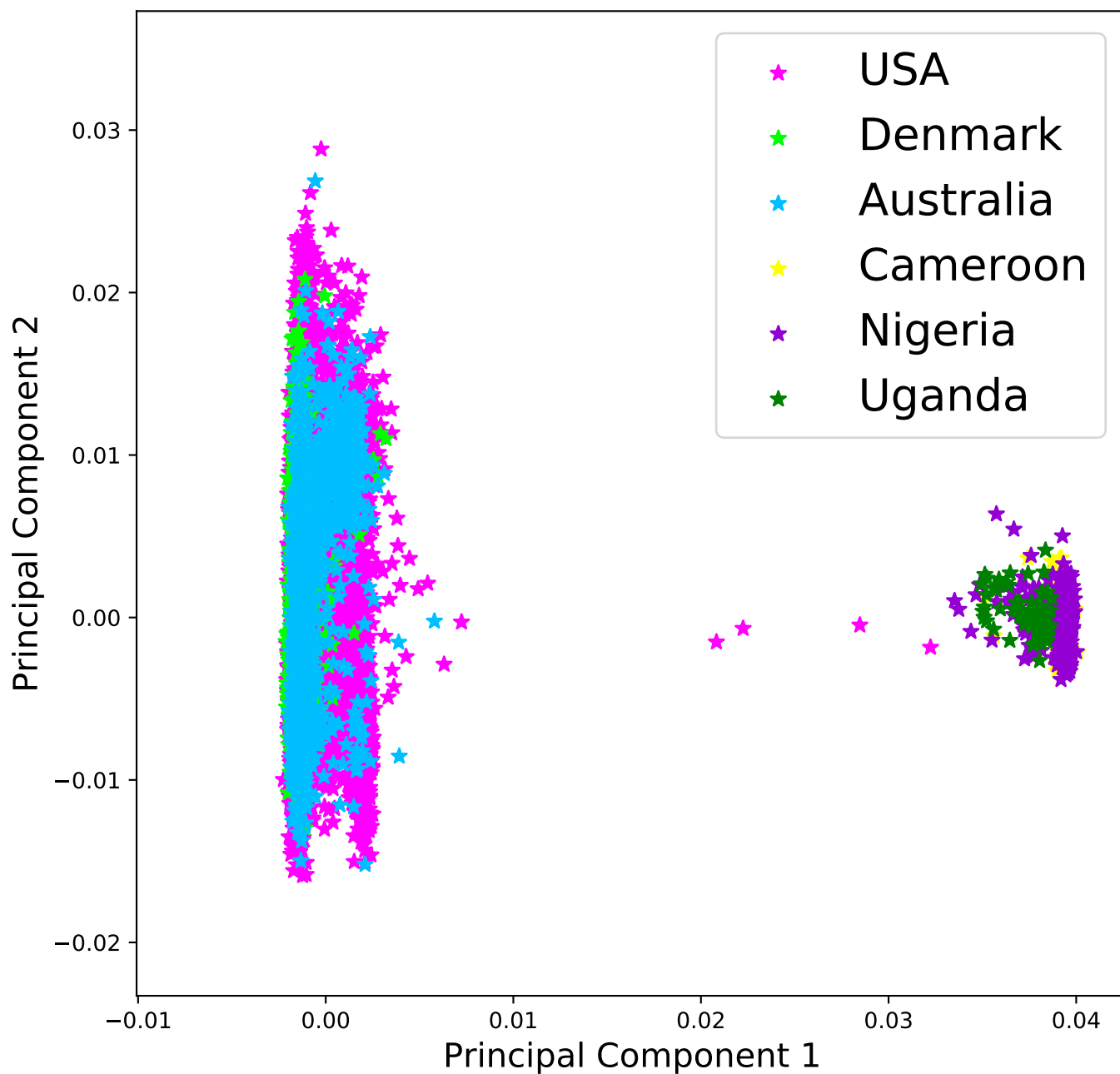

Supplement: S2 Fig — (PDF) [file pcbi.1012734.s004.pdf]

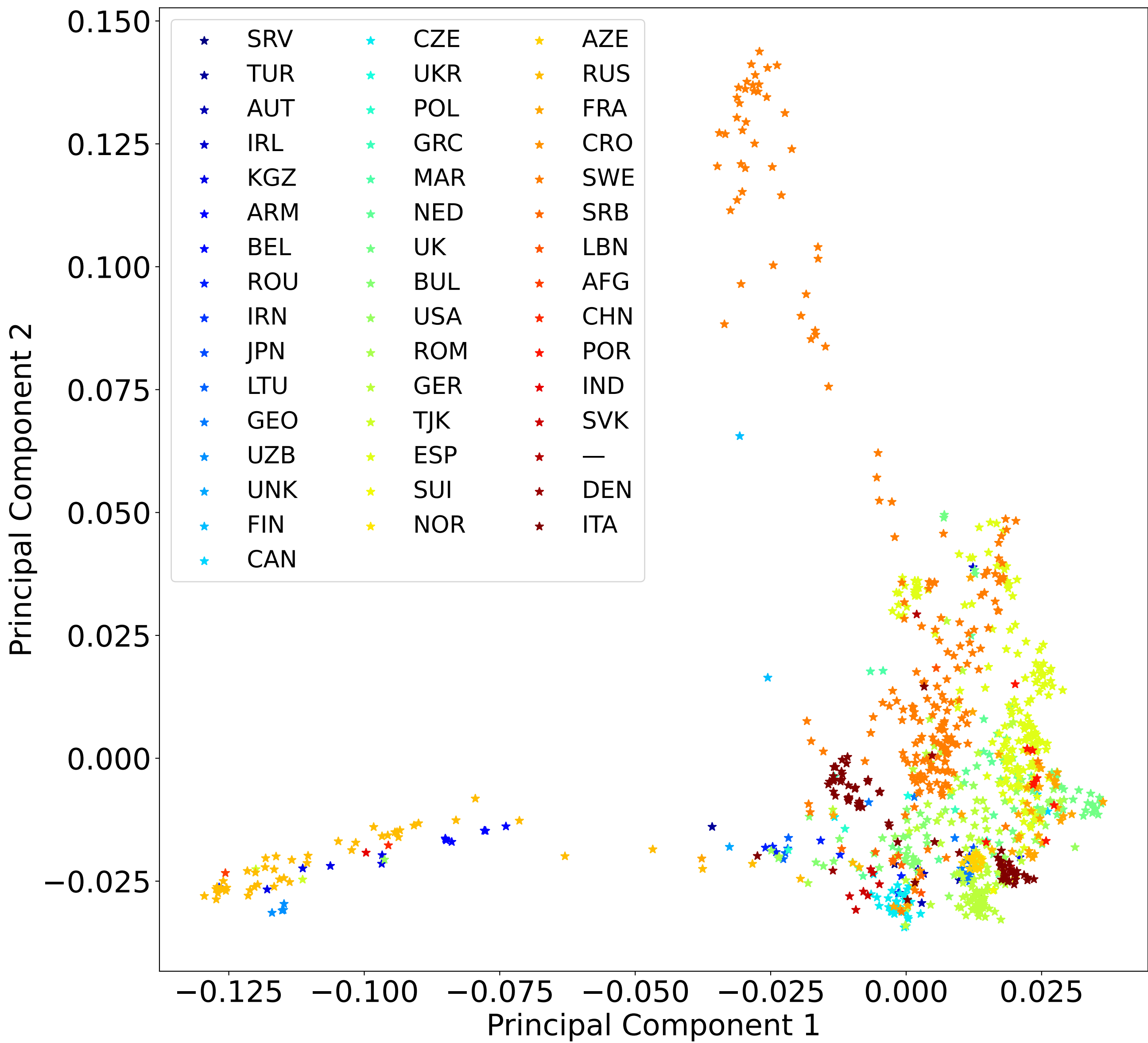

Supplement: S3 Fig — Projection of the Arabidopsis thaliana genotypes on the first two PCA components. Samples originate from 44 countries. (PDF) [file pcbi.1012734.s005.pdf]

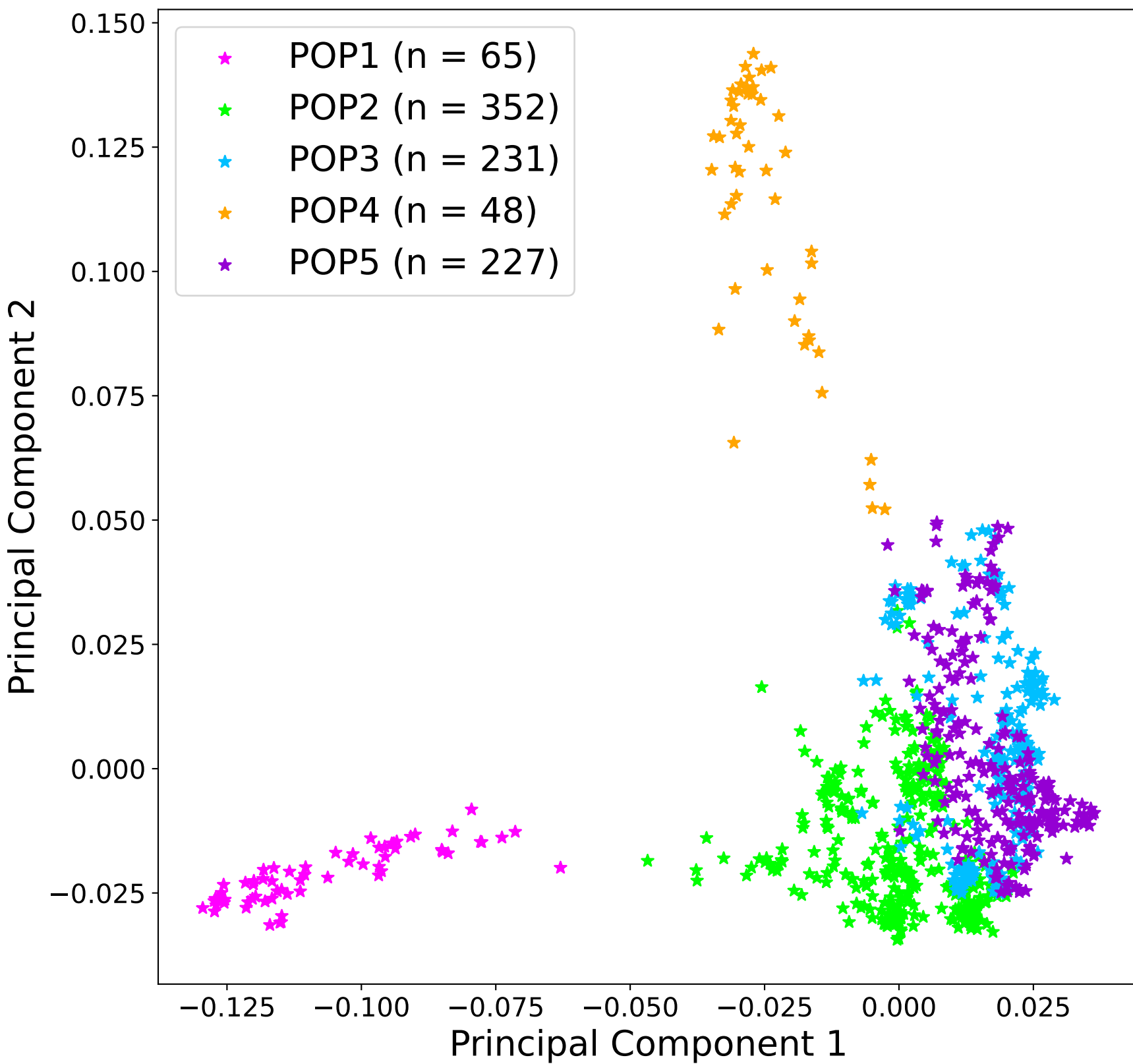

Supplement: S4 Fig — Projection of the Arabidopsis thaliana. The identified 5 subpopulations through K-means clustering of the data. (PDF) [file pcbi.1012734.s006.pdf]

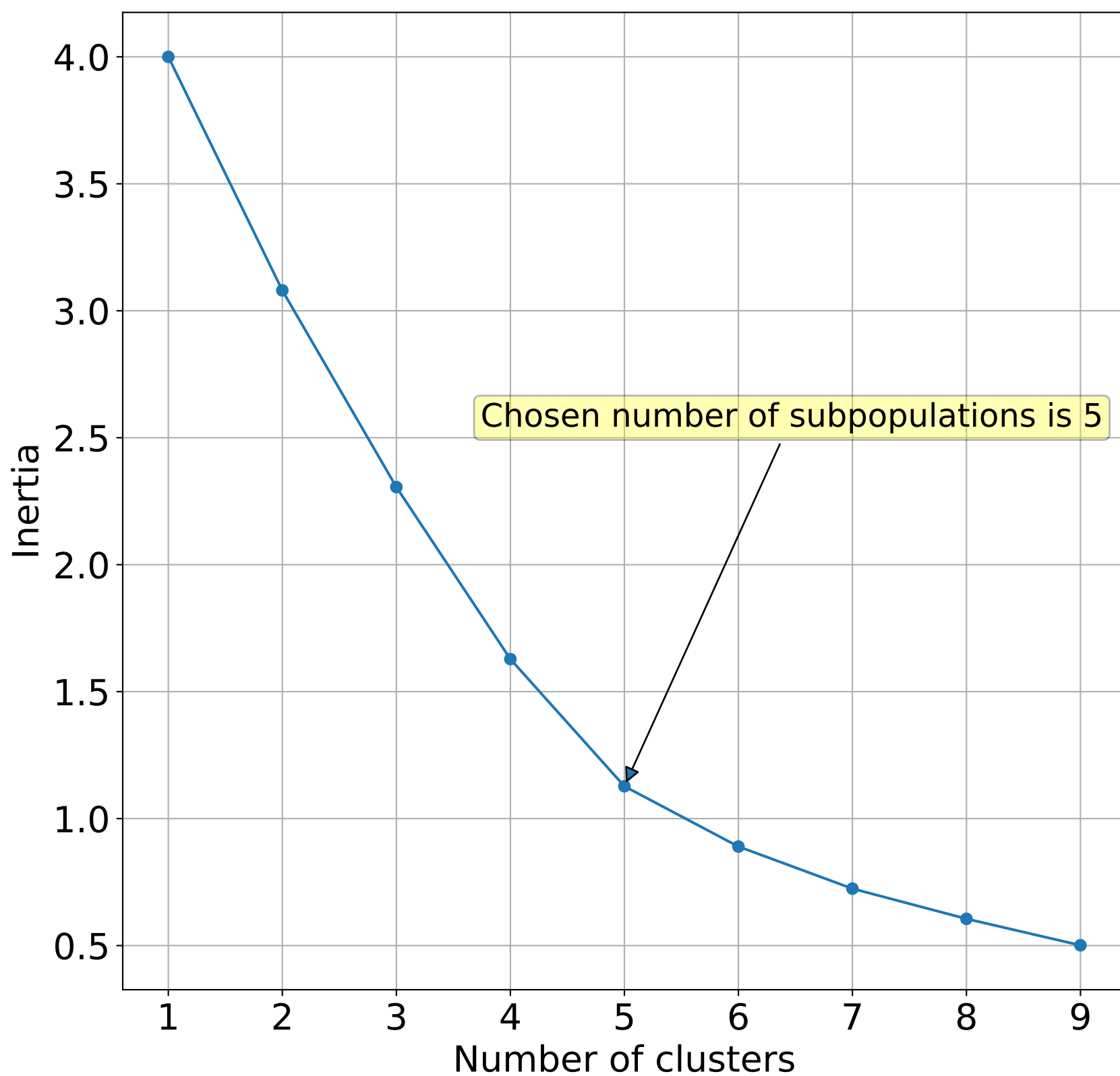

Supplement: S5 Fig — (PDF) [file pcbi.1012734.s007.pdf]

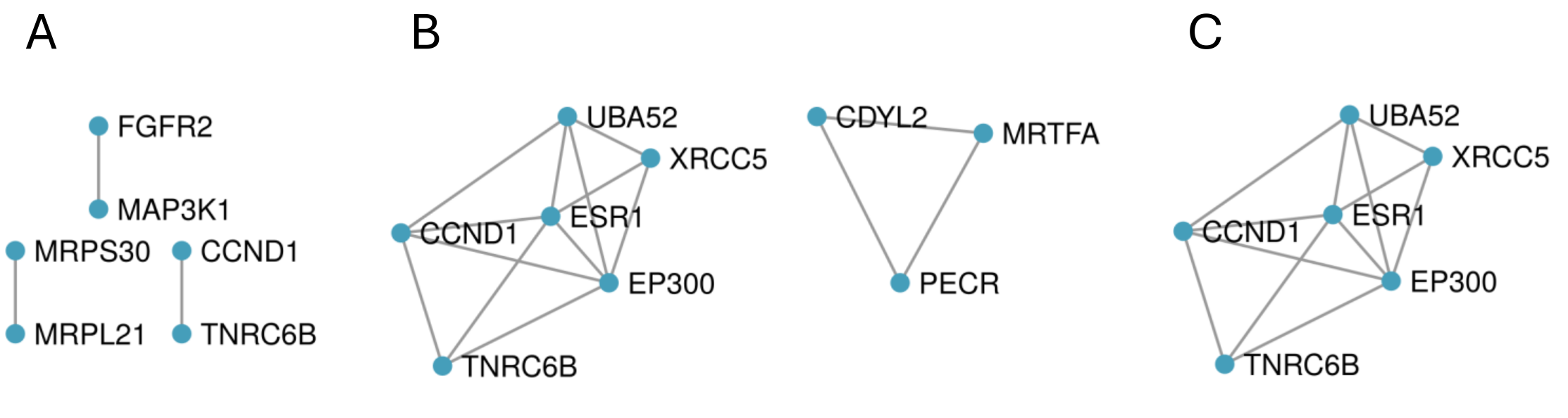

Supplement: S6 Fig — Modules of the PPI of known interactions between genes identified through physical and eQTL mapping of the SNPs selected by Adjusted GWAS, SMuGLasso and MuGLasso on DRIVE. (TIF) [file pcbi.1012734.s008.tif]

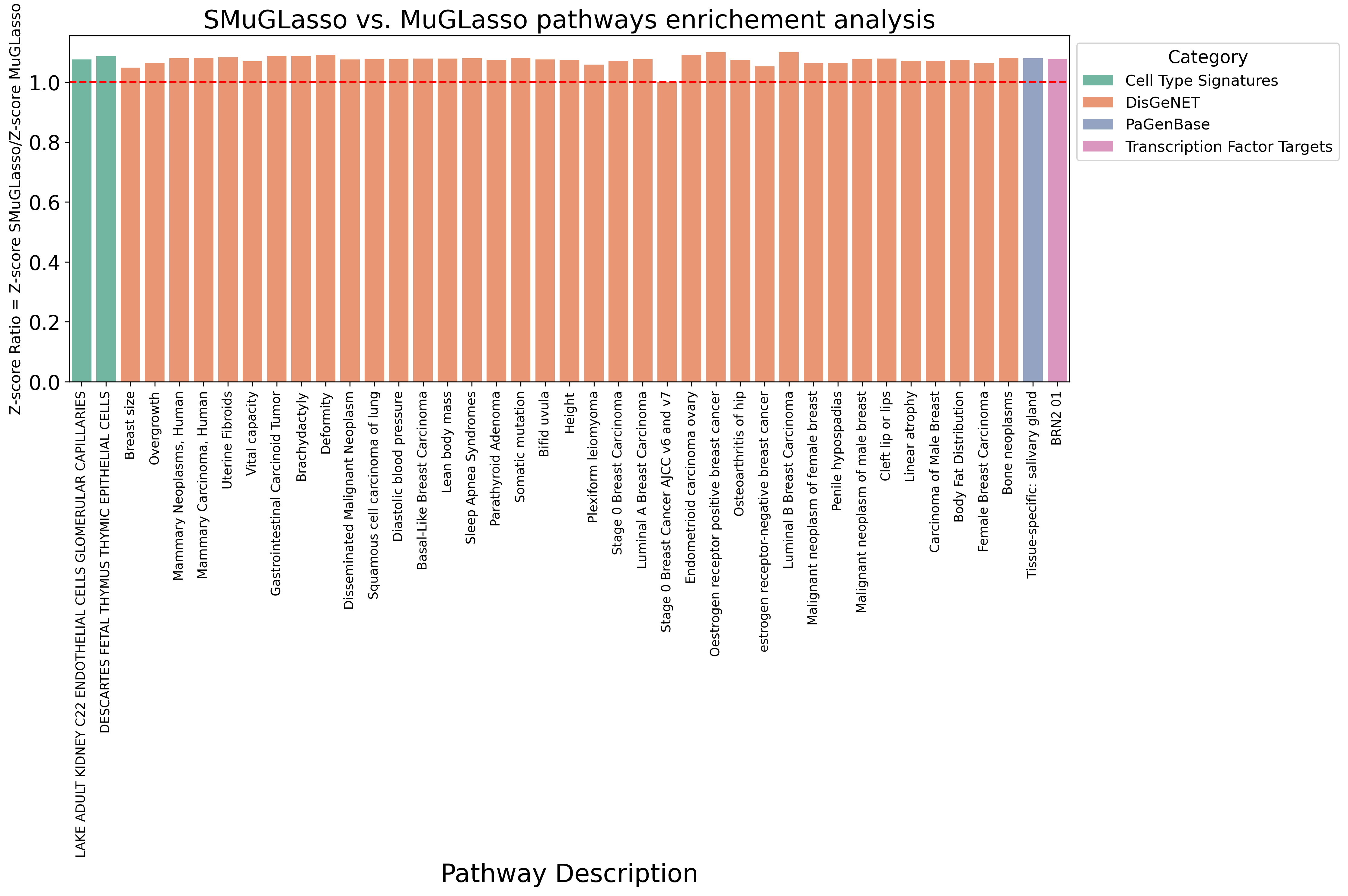

Supplement: S7 Fig — On DRIVE, comparison of All gene sets enrichment between SMuGLasso and MuGLasso based on Z-score ratios. Bar heights represent the ratio of Z-scores (SMuGLasso/MuGLasso) for top common gene sets. (PNG) [file pcbi.1012734.s009.png]

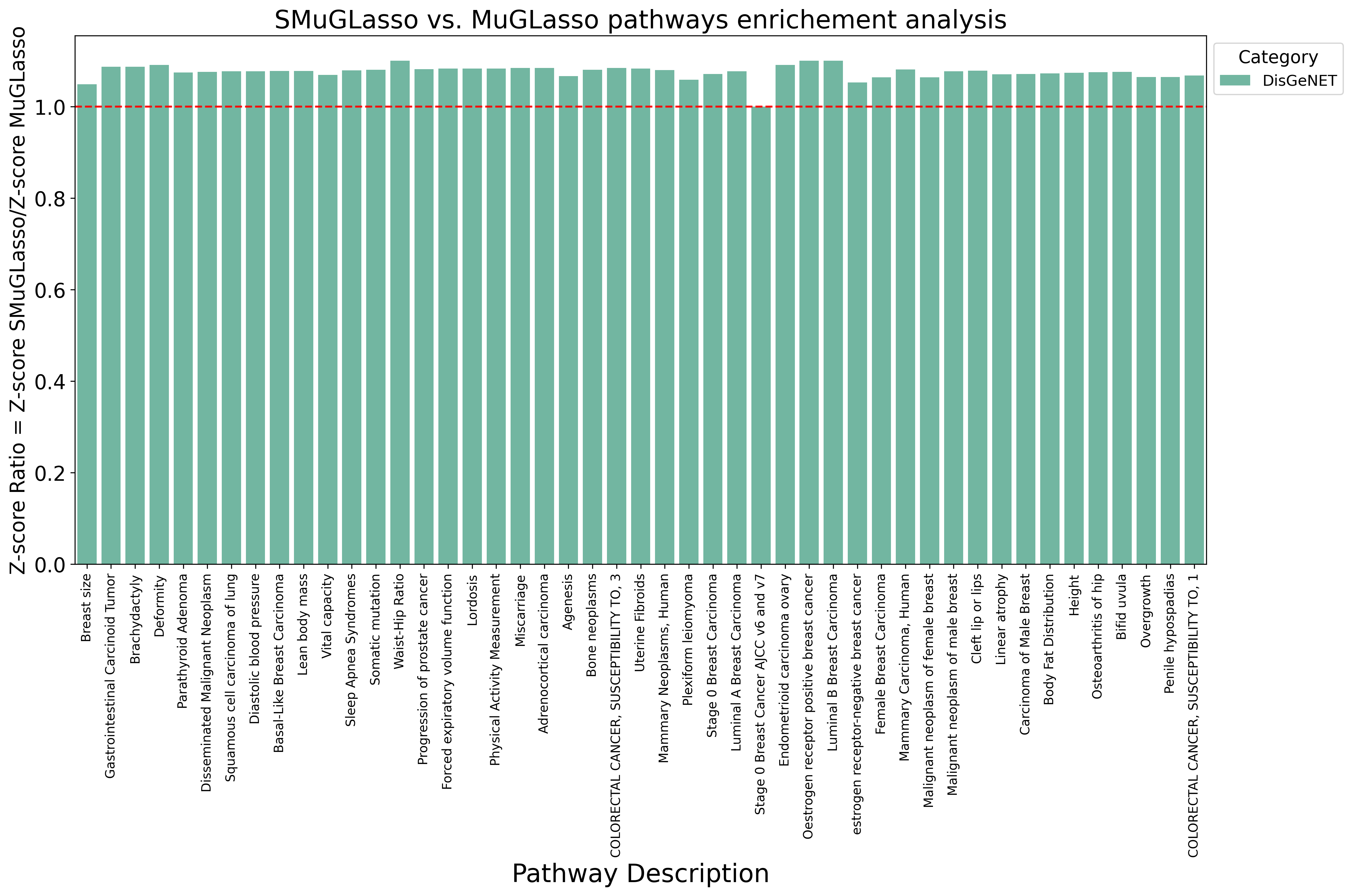

Supplement: S8 Fig — On DRIVE, comparison of DisGeNET gene sets enrichment between SMuGLasso and MuGLasso based on Z-score ratios. Bar heights represent the ratio of Z-scores (SMuGLasso/MuGLasso) for top DisGeNET common gene sets. (PNG) [file pcbi.1012734.s010.png]

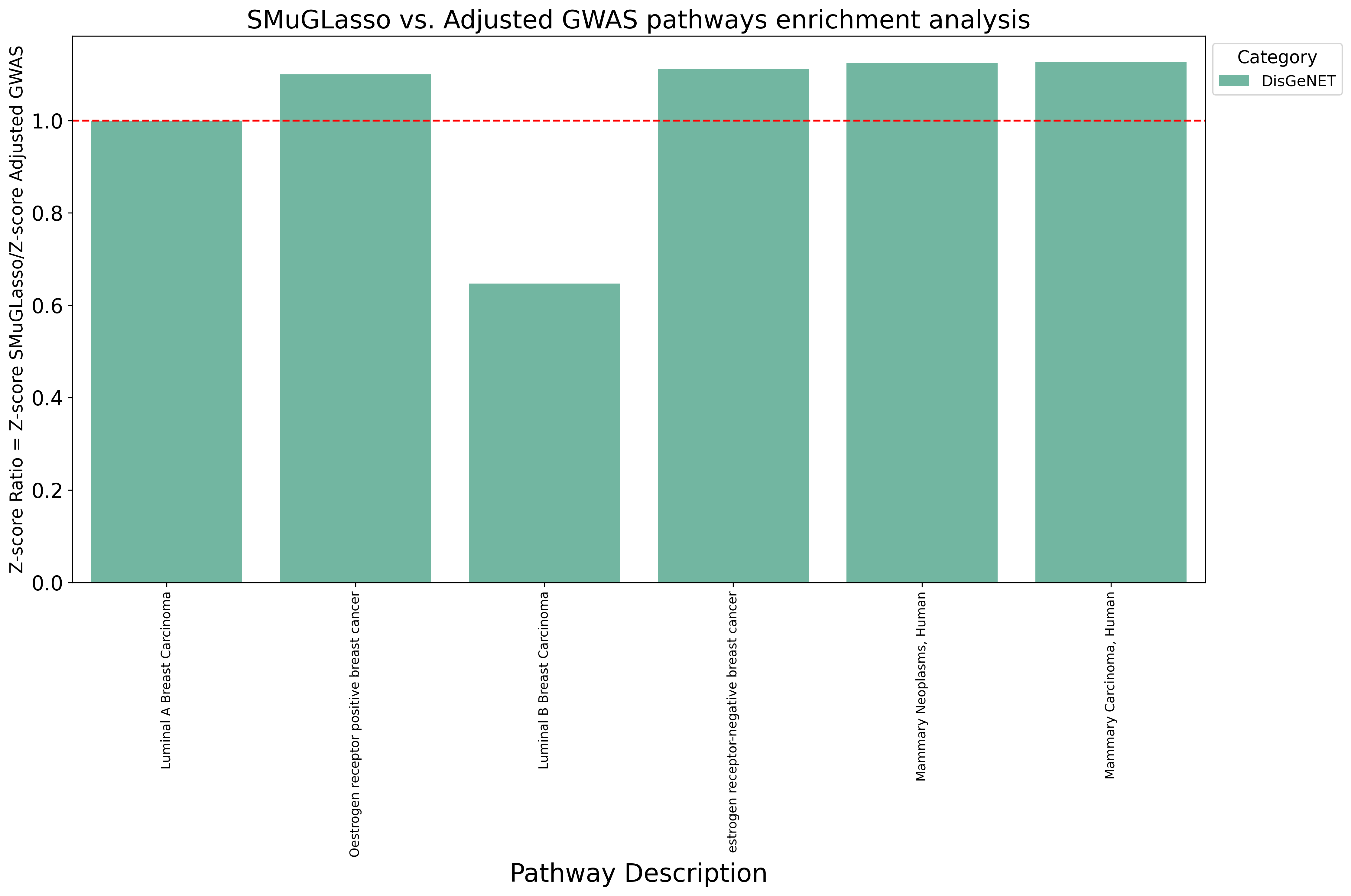

Supplement: S9 Fig — On DRIVE, comparison of DisGeNET gene sets enrichment between SMuGLasso and Adjusted GWAS based on Z-score ratios. Bar heights represent the ratio of Z-scores (SMuGLasso/Adjusted GWAS) for top DisGeNET common gene sets. (PNG) [file pcbi.1012734.s011.png]

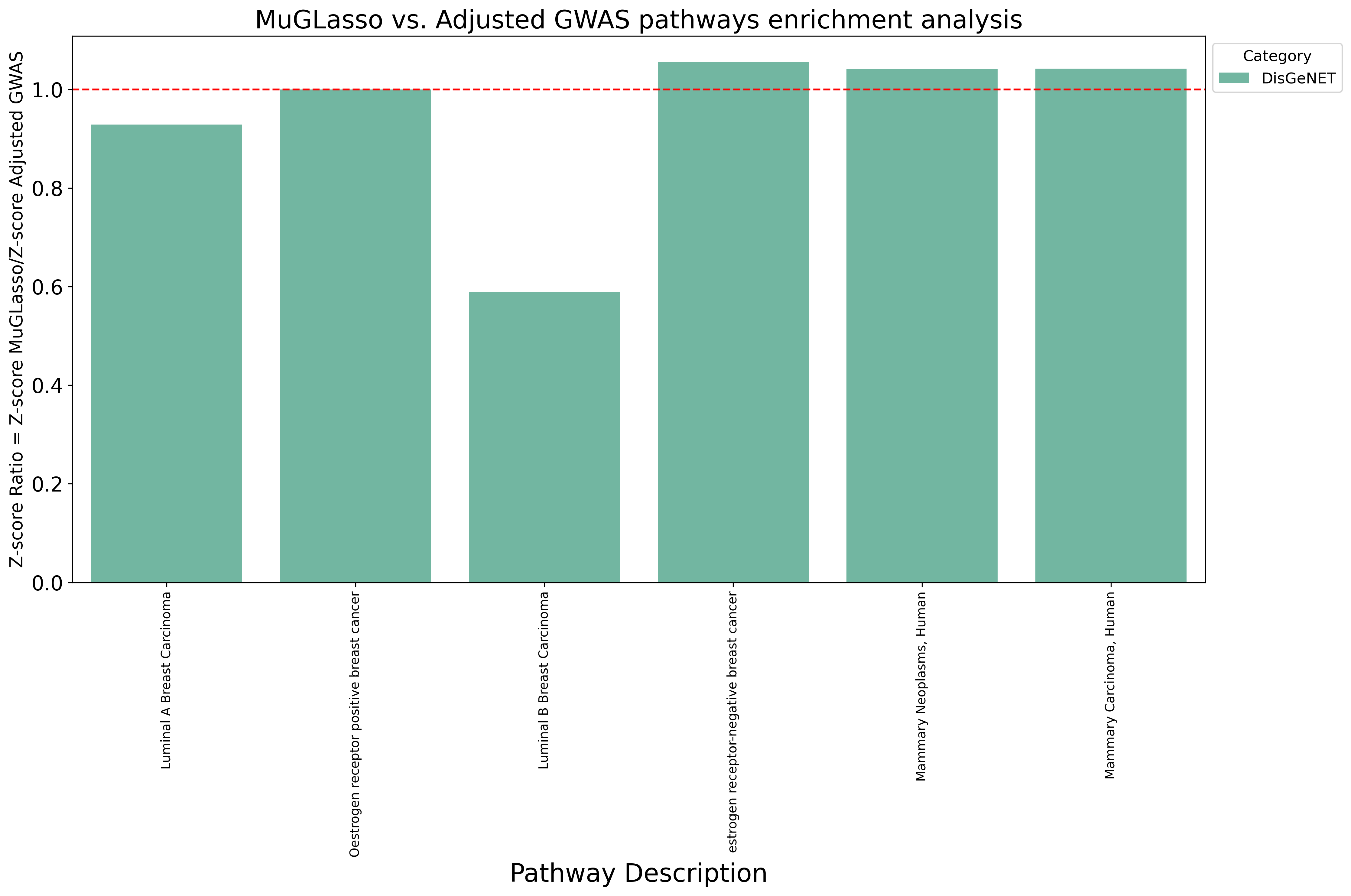

Supplement: S10 Fig — On DRIVE, comparison of DisGeNET gene sets enrichment between MuGLasso and Adjusted GWAS based on Z-score ratios. Bar heights represent the ratio of Z-scores (MuGLasso/Adjusted GWAS) for top DisGeNET common gene sets. (PNG) [file pcbi.1012734.s012.png]
