# Supplementary material for: Sparse multitask group Lasso for genome-wide association studies
Source: PLoS Comput Biol. 2025 Sep 12;21(9):e1012734. doi: 10.1371/journal.pcbi.1012734 (PMC12448984; doi:10.1371/journal.pcbi.1012734)
Supplement: S1 Table — (PDF) [file pcbi.1012734.s013.pdf]

S1 Table. Subpopulations of *Arabidopsis thaliana* with the corresponding countries and the number of samples included in each subpopulation.

Populations	Countries (# samples)
POP1	Russia(42), Armenia(7), Kyrgyzstan(5), Uzbekistan(4), Tajikistan(2), USA(1), India(1), Romania(1), China(1), Afghanistan(1)
POP2	Sweden(87), Germany(84), Italy(43), Czechia(32), Bulgaria(23), Russia(8), Romania(8), UK(7), Slovakia(7), Serbia(7), Austria(6), USA(6), France(6), Lithuania(4), Switzerland(3), Netherlands(3), Poland(3), Denmark(2), Japan(2), Croatia(2), Belgium(1), Finland(1), Norway(1), Greece(1), Lebanon(1), Turkey(1), North Vietnam(1), Unknown (1)
POP3	Spain(158), Italy(20), Azerbaijan(16), Georgia(14), Portugal(8), Bulgaria(5), Maroc(2), Greece(2), France(2), Ireland(1), Lebanon(1), Unknown(1)
POP4	Sweden(46), Finland(1)
POP5	Sweden(67), UK(43), France(32), Germany(28), USA(27), Italy(9), Spain(9), Netherlands(7), Canada(2), Ireland(1), Switzerland(1), Portugal(1)
