# Supplementary material for: Sparse multitask group Lasso for genome-wide association studies
Source: PLoS Comput Biol. 2025 Sep 12;21(9):e1012734. doi: 10.1371/journal.pcbi.1012734 (PMC12448984; doi:10.1371/journal.pcbi.1012734)
Supplement: S2 Table — CEU-specific selected genes are highlighted in blue and YRI-specific selected genes are highlighted in red. The remaining genes (in black) are risk genes shared across all populations. (PDF) [file pcbi.1012734.s014.pdf]

S2 Table. Potential breast cancer risk genes identified through physical (within 10kb) mapping of the loci selected by Adjusted GWAS, SMuGLasso and MuGLasso. CEU-specific selected genes are highlighted in blue and YRI-specific selected genes are highlighted in red. The remaining genes (in black) are risk genes shared across all populations.

Adjusted GWAS	**ITPR1**, **MRPS30**, **MAP3K1**, **SETD9**, **MIER3**, **EBF1**, **FGFR2**, **TOX3**, **MKL1**.
SMuGLasso	**ITPR1**, **MRPS30**, **MAP3K1**, **SETD9**, **MIER3**, **EBF1**, **FGFR2**, **TOX3**, **MKL1**, ADSL, ASTN2, CACNA1I, CCDC170, CCDC91, CDYL2, DIRC3, ELL, ESR1, FTO, GRHL1, HK1, HRSP12, KCNU1, NEK10, NUP205, PAX9, PTHLH, POP1, PPFIBP1, REP15, SGSM3, SSBP4, TGFBR2, TNRC6B, ZMIZ1, ZNF365.
MuGLasso	**ITPR1**, **MRPS30**, **MAP3K1**, **SETD9**, **MIER3**, **EBF1**, **FGFR2**, **TOX3**, **MKL1**, ADSL, ASTN2, C7orf73, CACNA1I, CCDC170, CCDC91, CCSER1, CD2AP, CDYL2, DIRC3, ELL, ESR1, FTO, GRHL1, HK1, HRSP12, KCNU1, LUC7L3, MED21, NEK10, NUP205, PAX9, PTHLH, POP1, PPFIBP1, REP15, SGSM3, SSBP4, TGFBR2, TNRC6B, ZMIZ1, ZNF365.
