# Supplementary material for: Sparse multitask group Lasso for genome-wide association studies
Source: PLoS Comput Biol. 2025 Sep 12;21(9):e1012734. doi: 10.1371/journal.pcbi.1012734 (PMC12448984; doi:10.1371/journal.pcbi.1012734)
Supplement: S3 Table — Potential breast cancer risk genes identified through both physical (within 10 kb) and eQTL mapping of the loci selected by MuGLasso or/and SMuGLasso and not the adjusted GWAS, found in meta-GWAS including the samples used in this work. (PDF) [file pcbi.1012734.s015.pdf]

S3 Table. MuGLasso or/and SMuGLasso specific Genes in Meta-GWAS via Physical and eQTL Mapping
Potential breast cancer risk genes identified through both physical (within 10kb) and eQTL mapping of the loci selected by MuGLasso or/and SMuGLasso and not the adjusted GWAS, found in meta-GWAS including the samples used in this work.

Gene symbols	Evidence
ASTN2	[1]
CCDC170	[2, 3, 4, 1, 5]
CDYL2	[3, 4, 1]
DIRC3	[3, 4, 1, 5]
ELL	[3, 4, 1, 5]
ESR1	[2, 4, 1, 5]
FTO	[2, 3, 4, 1, 5]
GRHL1	[1]
KCNU1	[4, 1]
NEK10	[3, 4, 1, 5]
PAX9	[3, 4, 1]
PTHLH	[2, 3, 4, 1, 5]
SSBP4	[1]
TGFBR2	[3, 4, 1]
TNRC6B	[1]
ZMIZ1	[3, 4, 1]
ZNF365	[1, 5]

References

[1] Michailidou K, Lindström S, et al. Association analysis identifies 65 new breast cancer risk loci. Nature. 2017;.

[2] Garcia-Closas M, Couch FJ, et al. Genome-wide association studies identify four ER negative-specific breast cancer risk loci. Nat Genet. 2013;.

[3] Michailidou K, Hall P, et al. Large-scale genotyping identifies 41 new loci associated with breast cancer risk. Nat Genet. 2013;.

[4] Michailidou K, Beesley J, et al. Genome-wide association analysis of more than 120,000 individuals identifies 15 new susceptibility loci for breast cancer. Nat Genet. 2015;.

[5] Milne RL, Kuchenbaecker KB, et al. Identification of ten variants associated with risk of estrogen-receptor-negative breast cancer. Nature Genetics. 2017;.
