# Supplementary material for: Sparse multitask group Lasso for genome-wide association studies
Source: PLoS Comput Biol. 2025 Sep 12;21(9):e1012734. doi: 10.1371/journal.pcbi.1012734 (PMC12448984; doi:10.1371/journal.pcbi.1012734)
Supplement: S4 Table — Potential breast cancer risk genes identified through both physical (within 10 kb) and eQTL mapping of the loci selected by MuGLasso or/and SMuGLasso and not the adjusted GWAS, found to be associated with breast cancer risk or tumor growth in the literature. (PDF) [file pcbi.1012734.s016.pdf]

S4 Table. MuGLasso or/and SMuGLasso specific Genes Linked to Breast Cancer in Literature
Potential breast cancer risk genes identified through both physical (within 10kb) and eQTL mapping of the loci selected by MuGLasso or/and SMuGLasso and not the adjusted GWAS, found to be associated with breast cancer risk or tumor growth in the literature.

Gene symbols	Evidence
ADSL	oncogenic driver in triple negative breast cancer [1]
CACNA1I	underexpressed in breast cancer [2]
CCDC91	likely target gene of breast cancer risk variants [3]
NUP205	forms a complex with NUP93 which regulates breast tumor growth [4]
POP1	expression correlates with prognosis in breast cancer [5]
PPFIBP1	promotes cell motility and migration in breast cancer [6]
SGSM3	associated with breast cancer in a Chinese population [7]
HK1	promote breast cancer cell proliferation, migration and invasion [8]
LUC7L3	targeted by miR-370-5p, suppresses proliferation and invasion in breast cancer cells [9]
Other genes	
C7orf73, *CCSER1*, *CD2AP*, *HRSP12*, *MED21*, *REP15*	

References

[1] Zurlo G, Liu X, et al. Prolyl hydroxylase substrate adenylosuccinate lyase is an oncogenic driver in triple negative breast cancer. Nature Communications. 2019;.

[2] Phan NN, Wang CY, et al. Voltage-gated calcium channels: Novel targets for cancer therapy. Oncol Lett. 2017;.

[3] Ferreira MA, Gamazon ER, et al. Genome-wide association and transcriptome studies identify target genes and risk loci for breast cancer. Nature Communications. 2019;.

[4] Bersini S, Lytle NK, et al. Nup93 regulates breast tumor growth by modulating cell proliferation and actin cytoskeleton remodeling. Life Sci Alliance. 2020;.

[5] Liu Y, Sun H, et al. Identification of a Three-RNA Binding Proteins (RBPs) Signature Predicting Prognosis for Breast Cancer. Front Oncol. 2021;.

[6] Chiaretti S, Astro V, et al. Effects of the scaffold proteins liprin-α1, β1 and β2 on invasion by breast cancer cells. Biol Cell. 2016;.

[7] Tan T, Zhang K, Chen W. Genetic variants of ESR1 and SGSM3 are associated with the susceptibility of breast cancer in the Chinese population. Breast Cancer. 2017;.

[8] Ma X, Chen J, et al. ErbB2-upregulated HK1 and HK2 promote breast cancer cell proliferation, migration and invasion. Med Oncol. 2023;.

[9] Sang K, Yi T, et al. MiR-370-5p inhibits the progression of breast cancer via targeting LUC7L3. J Recept Signal Transduct Res. 2020;.
