# Supplementary material for: Sparse multitask group Lasso for genome-wide association studies
Source: PLoS Comput Biol. 2025 Sep 12;21(9):e1012734. doi: 10.1371/journal.pcbi.1012734 (PMC12448984; doi:10.1371/journal.pcbi.1012734)
Supplement: S5 Table — Genes identified through physical mapping of SNPs selected as associated with flowering time in Arabidopsis thaliana using SMuGLasso, MuGLasso and Adjusted GWAS. (PDF) [file pcbi.1012734.s017.pdf]

S5 Table. DTF3 loci detected by SMuGLasso and MuGLasso on *Arabidopsis thaliana* dataset. Genes identified through physical mapping of SNPs selected as associated with flowering time in *Arabidopsis thaliana* using SMuGLasso, MuGLasso and Adjusted GWAS.

Methods	List of selected genes
Adjusted GWAS	**AT5G10100**, **AT5G45830**, **AT4G00730**, **AT4G00752**, **AT4G00630**, **AT4G00740**, **AT4G00750**.
SMuGLasso	**AT5G10100**, **AT5G45830**, **AT4G00730**, **AT4G00752**, **AT4G00630**, **AT4G00740**, **AT4G00750**, AT4G01915, AT5G15020, AT5G17710, AT5G27945, AT5G53410, AT3G58590, AT1G20130, AT3G29450, AT3G14490, AT1G03365, AT3G14470, AT1G28410, AT2G23430, AT3G27040, AT4G17970, AT4G09160, AT2G34890, AT4G30100, AT2G39990, AT4G35080, AT2G18500, AT3G46340, AT1G29300, AT3G27670, AT5G41820, AT2G38720, AT3G44610, AT4G33760, AT5G40450, AT1G27520, AT3G26140, AT4G16990, AT1G61360, AT3G61170, AT5G55910, AT2G25940, AT5G51830, AT1G43600, AT2G39310, AT4G34310, AT1G78970.
MuGLasso	**AT5G10100**, **AT5G45830**, **AT4G00730**, **AT4G00752**, **AT4G00630**, **AT4G00740**, **AT4G00750**, AT4G01915, AT5G15020, AT5G17710, AT5G27945, AT5G53410, AT3G58590, AT1G20130, AT3G29450, AT3G14490, AT1G03365, AT3G14470, AT1G28410, AT2G23430, AT3G27040, AT4G17970, AT4G09160, AT2G34890, AT4G30100, AT2G39990, AT4G35080, AT2G18500, AT3G46340, AT1G29300, AT3G27670, AT5G41820, AT2G38720, AT3G44610, AT4G33760, AT5G40450, AT1G27520, AT3G26140, AT4G16990, AT1G61360, AT1G43600, AT2G39310, AT4G34310, AT1G78970, AT4G33480, AT5G40290, AT1G12970, AT3G13550, AT2G32170, AT4G27290, AT1G59690, AT3G61170 AT5G55910, AT2G25940, AT5G51830.
