# Supplementary material for: Sparse multitask group Lasso for genome-wide association studies
Source: PLoS Comput Biol. 2025 Sep 12;21(9):e1012734. doi: 10.1371/journal.pcbi.1012734 (PMC12448984; doi:10.1371/journal.pcbi.1012734)
Supplement: S6 Table — Potential breast cancer risk genes identified through both physical (within 10 kb) and eQTL mapping of the loci selected by Adjusted GWAS, SMuGLasso and MuGLasso. CEU-specific selected genes are highlighted in blue and YRI-specific selected genes are highlighted in red. The remaining genes (in black) are risk genes shared across all populations. (PDF) [file pcbi.1012734.s018.pdf]

S6 Table. Breast cancer risk loci detected by SMuGLasso and MuGLasso on DRIVE
Potential breast cancer risk genes identified through both physical (within 10kb) and eQTL mapping of the loci selected by Adjusted GWAS, SMuGLasso and MuGLasso. CEU-specific selected genes are highlighted in blue and YRI-specific selected genes are highlighted in red. The remaining genes (in black) are risk genes shared across all populations.

Adjusted GWAS	**ITPR1**, **MRPS30**, **MAP3K1**, **SETD9**, **MIER3**, **EBF1**, **FGFR2**, **TOX3**, **MKL1**, **PDE4DIP**, **ANKRD55**, **NRIP1**, **XPNPEP3**, **CCND1**, **FAM72B**, **TCF20**, **MCHR1**, **AC022431.2**, **HCN1**, **CHADL**, **CHMP6**, **PARP8**, **USP25**, **TNRC6B**, **AL590452.1**, **REG4**, **L3MBTL2**, **MRPL21**, **IGFBP2**, **AL592284.1**, **NBPF14**, **PTHLH**, **NNT**, **STARD5**.
SMuGLasso	**ITPR1**, **MRPS30**, **MAP3K1**, **SETD9**, **MIER3**, **EBF1**, **FGFR2**, **TOX3**, **MKL1**, **PDE4DIP**, **ANKRD55**, **NRIP1**, **XPNPEP3**, **CCND1**, **FAM72B**, **TCF20**, **MCHR1**, **AC022431.2**, **HCN1**, **CHADL**, **CHMP6**, **PARP8**, **USP25**, **TNRC6B**, **AL590452.1**, **REG4**, **L3MBTL2**, **MRPL21**, **IGFBP2**, **AL592284.1**, **NBPF14**, **PTHLH**, **NNT**, **STARD5**, ADSL, ASTN2, CACNA1I, CCDC170, CCDC91, CDYL2, DIRC3, ELL, ESR1, FTO, GRHL1, HK1, HRSP12, KCNU1, NEK10, NUP205, PAX9, POP1, PPFIBP1, REP15, SGSM3, SSBP4, TGFBR2, ZMIZ1, ZNF365, KLF11, KLF4, RNF145, CNOT4, ZNF703, MAT2A, UTP18, UBA52, GPR115, ACVR1B, POLR3H, CRTC1, MGAT3, PHGDH, GPR111, SLC4A7, TAF1B, EP300, ACOT4, ABCC3, XRCC5, ISYNA1, HOMER3, PLEKHA1, SLC38A9, TACR2, LRRC25, SPATA20, GDF15, SUMF1.
MuGLasso	**ITPR1**, **MRPS30**, **MAP3K1**, **SETD9**, **MIER3**, **EBF1**, **FGFR2**, **TOX3**, **MKL1**, **PDE4DIP**, **ANKRD55**, **NRIP1**, **XPNPEP3**, **CCND1**, **FAM72B**, **TCF20**, **MCHR1**, **AC022431.2**, **HCN1**, **CHADL**, **CHMP6**, **PARP8**, **USP25**, **TNRC6B**, **AL590452.1**, **REG4**, **L3MBTL2**, **MRPL21**, **IGFBP2**, **AL592284.1**, **NBPF14**, **PTHLH**, **NNT**, **STARD5**, ADSL, ASTN2, C7orf73, CACNA1I, CCDC170, CCDC91, CCSER1, CD2AP, CDYL2, DIRC3, ELL, ESR1, FTO, GRHL1, HK1, HRSP12, KCNU1, LUC7L3, MED21, NEK10, NUP205, PAX9, POP1, PPFIBP1, REP15, SGSM3, SSBP4, TGFBR2, ZMIZ1, ZNF365, SAR1A, KLF11, KLF4, PECR, ST13, RNF145, CNOT4, ZNF703, MAT2A, UTP18, UBA52, GPR115, HKDC1, ACVR1B, POLR3H, CRTC1, MGAT3, RP11-863K10.7, PHGDH, GPR111, SLC4A7, TMEM59L, TAF1B, EP300, ANKRD40, CRLF1, ACOT4, ABCC3, XRCC5, ISYNA1, HOMER3, PLEKHA1, KXD1, SLC13A4, C19orf60, PGPEP1, SLC38A9, TACR2, LRRC25, SPATA20, GDF15, SUMF1.
