# Supplementary material for: Sparse multitask group Lasso for genome-wide association studies
Source: PLoS Comput Biol. 2025 Sep 12;21(9):e1012734. doi: 10.1371/journal.pcbi.1012734 (PMC12448984; doi:10.1371/journal.pcbi.1012734)
Supplement: S7 Table — The potential breast cancer risk genes within 10 kb of loci obtained through eQTL analysis, identified by MuGLasso or/and SMuGLasso and not the adjusted GWAS, found to be associated with breast cancer risk or tumor growth in the literature. (PDF) [file pcbi.1012734.s019.pdf]

S7 Table. MuGLasso or/and SMuGLasso specific eQTL Genes linked to Breast Cancer

The potential breast cancer risk genes within 10kb of loci obtained through eQTL analysis, identified by MuGLasso or/and SMuGLasso and not the adjusted GWAS, found to be associated with breast cancer risk or tumor growth in the literature.

Gene symbols	Evidence
KLF11	tumor suppressor, inhibiting cell proliferation and promoting apoptosis [1, 2]
KLF4	promotes the population of cancer stem cells and enhances cell migration and invasion [3]
CNOT4	aids YAP degradation, inhibiting triple-negative breast cancer via hydnocarpin [4, 5]
SUMF1	its high expression reduce median survival in ER-negative breast cancer patient [6]
UBA52	associated with Lapatinib resistance in breast cancer [7]
ACVR1B	exhibit varied expression in breast cancer subtypes linked to bone morphogenetic proteins [8]
CRTC1	regulates aromatase activity in primary human breast preadipocytes and estrogen [9]
MAT2A	correlates with poorer survival in breast cancer patients and increased invasiveness of cancer cells [10]
ST13	associated with HSP70 protein interaction, shows frequent loss of heterozygosity in breast cancer [11]
RNF145	tumor suppressor in breast cancer through ubiquitin ligase activity-dependent regulation [12]
ZNF703	promotes triple-negative breast cancer cells through cell-cycle signaling and associated with poor prognosis [13]
UTP18	acts as a prognostic indicator for breast cancer [14]
HKDC1	promotes tumor growth and metastasis through the PGC1β/SREBP1 pathway [15]
MGAT3	associated with breast cancer through its role in glycosylation, influencing tumor behavior and patient outcomes [16]
PHGDH	promotes breast cancer metastasis through non-catalytic mechanisms involving altered protein glycosylation [17]
GPR111, GPR115	promote breast cancer cell migration and invasion [18]
SLC4A7	enhance tumor growth and development of breast cancer [19]
TAF1A	breast cancer progression from early to advanced stages [20]
EP300	promotes cancer stem cell traits and metastasis in triple-negative and basal-like breast cancer [21]
ABCC3	contributes to breast cancer chemoresistance [22]
XRCC5	associated with an increased risk of breast cancer [23]
ISYNA1	its Hypomethylation in peripheral blood is associated with an increased risk of breast cancer in the Chinese population. [24]
HOMER3	promotes metastasis in triple-negative breast cancer [25]
SLC38A9	regulates mTORC1 activity, which is often overactive in breast cancer [26]
LRRC25	associated with an increased risk of breast cancer [27]
GDF15	associated with paclitaxel resistance and unfavorable prognosis in triple-negative breast cancer [28]
TACR2	its expression alterations is linked to breast cancer [29]
Other genes	
POLR3H, *KXD1*, *RP11-863K10.7*, *CRLF1*, *PECR*, *TMEM59L*, *PLEKHA1*, *SAR1A*, *ANKRD40*, *ACOT4*, *SLC13A4*, *C19orf60*, *PGPEP1*, *SPATA20*	

References

[1] Lin L, et al. The Distinct Roles of Transcriptional Factor KLF11 in Normal Cell Growth Regulation and Cancer as a Mediator of TGF-β Signaling Pathway. Int J Mol Sci. 2020;.

[2] Lin L, et al. KLF11 is an independent negative prognostic factor for breast cancer from a cohort study and induces proliferation and inhibits apoptosis in vitro. Breast Cancer. 2023;.

[3] Yu F, et al. Kruppel-like factor 4 (KLF4) is required for maintenance of breast cancer stem cells and for cell migration and invasion. Oncogene. 2011;.

[4] Ou HL, et al. Hydnocarpin inhibits malignant progression of triple negative breast cancer via CNOT4-mediated ubiquitination and degradation of YAP. Zhongguo Zhong Yao Za Zhi. 2023;.

[5] Wang Y, et al. TNKS1BP1 facilitates ubiquitination of CNOT4 by TRIM21 to promote hepatocellular carcinoma progression and immune evasion. Cell Death and Disease. 2024;.

[6] Kumari K, et al. miRNA-mediated alteration of sulfatase modifying factor 1 expression using self-assembled branched DNA nanostructures. RSC Adv. 2021;.

[7] Zhang L, et al. Identification and characterization of biomarkers and their-functions for Lapatinib-resistant breast cancer. Med Oncol. 2017;.

[8] Liu M, et al. Aberrant expression of bone morphogenetic proteins in the disease progression and metastasis of breast cancer. Front Oncol. 2023;.

[9] Samarajeewa NU, et al. CREB-Regulated Transcription Co-Activator Family Stimulates Promoter II-Driven Aromatase Expression in Preadipocytes. Horm Cancer. 2013;.

[10] Chu PY, et al. MAT2A Localization and Its Independently Prognostic Relevance in Breast Cancer Patients. Int J Mol Sci. 2021;.

[11] BAI R, et al. ST13, a proliferation regulator, inhibits growth andmigration of colorectal cancer cell lines. Journal of Zhejiang University-SCIENCE B. 2012;.

[12] Yang YL, et al. RNF144A functions as a tumor suppressor in breast cancer through ubiquitin ligase activity-dependent regulation of stability and oncogenic functions of HSPA2. Cell Death Differ 27. 2019;.

[13] Zhang X, et al. ZNF703 promotes triple-negative breast cancer cells through cell-cycle signaling and associated with poor prognosis. BMC Cancer. 2022;.

[14] Yang H, et al. A small subunit processome protein promotes cancer byaltering translation. Oncogene. 2014;.

[15] Chen X, et al. PGC1β Regulates Breast Tumor Growth and Metastasis by SREBP1-Mediated HKDC1 Expression. Front Oncol. 2019;.

[16] Miwa HE, et al. Bisected, complex N-glycans and galectins in mouse mammary tumor progression and human breast cancer. Glycobiology. 2013;.

[17] Rossi M, et al. PHGDH heterogeneity potentiates cancer cell dissemination and metastasis. Nature. 2022;.

[18] Rosa M, et al. Emerging roles of adhesion G protein-coupled receptors. Biochem Soc Trans 27. 2021;.

[19] Lee S, et al. Na+,HCO3− -cotransporter NBCn1 (Slc4a7) accelerates ErbB2-induced breast cancer development and tumor growth in mice. Oncogene 37. 2018;.

[20] Rossetti S, et al. Mammary epithelial morphogenesis and early breast cancer. Evidence of involvement of basal components of the RNA Polymerase I transcription machinery. Cell Cycle. 2016;.

[21] Ring A, et al. EP300 knockdown reduces cancer stem cell phenotype, tumor growth and metastasis in triple negative breast cancer. BMC Cancer. 2020;.

[22] Balaji SA, et al. Role of the Drug Transporter ABCC3 in Breast Cancer Chemoresistance. PLoS One. 2016;.

[23] Rajaei M, et al. Association between polymorphisms at promoters of XRCC5 and XRCC6 genes and risk of breast cancer. Med Oncol. 2014;.

[24] Liu C, et al. The Association Between Breast Cancer and Blood-Based Methylation of CD160, ISYNA1 and RAD51B in the Chinese Population. Front Genet. 2022;.

[25] Liu Q, et al. HOMER3 facilitates growth factor-mediated β-Catenin tyrosine phosphorylation and activation to promote metastasis in triple negative breast cancer. J Hematol Oncol 14. 2021;.

[26] Hare SH, Harvey AJ. mTOR function and therapeutic targeting in breast cancer. Am J Cancer Res. 2017;.

[27] Hoffman JD, et al. Cis-eQTL-based trans-ethnic meta-analysis reveals novel genes associated with breast cancer risk. PLoS Genet. 2017;.

[28] He Y, et al. Growth differentiation factor 15 is required for triple-negative breast cancer cell growth and chemoresistance. Anticancer Drugs. 2023;.

[29] Sánchez ML, et al. Peptidergic Systems and Cancer: Focus on Tachykinin and Calcitonin/Calcitonin Gene-Related Peptide Families. Cancers (Basel). 2023;.
