# Supplementary material for: Sparse multitask group Lasso for genome-wide association studies
Source: PLoS Comput Biol. 2025 Sep 12;21(9):e1012734. doi: 10.1371/journal.pcbi.1012734 (PMC12448984; doi:10.1371/journal.pcbi.1012734)
Supplement: S8 Table — (PDF) [file pcbi.1012734.s020.pdf]

S8 Table. Stability index and number of selected features for different methods on *Arabidopsis thaliana*

Methods	# selected LD-groups	# selected SNPs	Stability index	Selection level
SMuGLasso	80	6 367	0.4315	LD-groups
SMuGLasso without stability selection	87	7 220	0.3883	LD-groups
MuGLasso	104	8 254	0.5733	LD-groups
MuGLasso without stability selection	149	10 935	0.5040	LD-groups
Adjusted group Lasso + stability selection	90	6 944	0.4489	LD-groups
Adjusted group Lasso	114	8 358	0.3654	LD-groups
Stratified group Lasso	133	10 135	0.3147	LD-groups
Adjusted Lasso	112	9 258	0.2600	Single-SNP
Stratified Lasso	135	9 897	0.2140	Single-SNP
Adjusted GWAS	7	31	0.9800	Single-SNP
FastLMM	5	12	0.9789	Single-SNP
