# Supplementary material for: Sparse multitask group Lasso for genome-wide association studies
Source: PLoS Comput Biol. 2025 Sep 12;21(9):e1012734. doi: 10.1371/journal.pcbi.1012734 (PMC12448984; doi:10.1371/journal.pcbi.1012734)
Supplement: S9 Table — “#” is the number of genes in the user-provided lists with membership in the given ontology term. “%” is the percentage of genes selected by SMuGLasso that are found in the given ontology term (only input genes with at least one ontology term annotation are included in the calculation). “Log10(P)” is the p-value in log base 10. “Log10(q)” is the multi-test adjusted p-value in log base 10. (PDF) [file pcbi.1012734.s021.pdf]

S9 Table. Summary of pathway and process enrichment analysis: Top 10 clusters of enriched terms, each described by one representative enriched term. "#" is the number of genes in the user-provided lists with membership in the given ontology term. "%" is the percentage of genes selected by SMuGLasso that are found in the given ontology term (only input genes with at least one ontology term annotation are included in the calculation). "Log10(P)" is the p-value in log base 10. "Log10(q)" is the multi-test adjusted p-value in log base 10.

GO	Category	Description	#	%	Log10 (P)	Log10 (q)	Gene Hits
GO:0060443	GO Biological Processes	mammary gland morphogenesis	3	8.33	-4.73	-0.39	ESR1, FGFR2, TGFBR2
R-HSA-9006925	Reactome Gene Sets	Intracellular signaling by second messengers	4	11.11	-3.31	0.00	ESR1, FGFR2, ITPR1, TNRC6B
WP2858	WikiPathways	Ectoderm differentiation	3	8.33	-3.19	0.00	FGFR2, TOX3, SGSM3
WP2853	WikiPathways	Endoderm differentiation	3	8.33	-3.17	0.00	PAX9, PTHLH, TOX3
R-HSA-3108232	Reactome Gene Sets	SUMO E3 ligases SUMOylate target proteins	3	8.33	-2.87	0.00	ESR1, NUP205, MRTFA
GO:0061014	GO Biological Processes	positive regulation of mRNA catabolic process	3	8.33	-2.81	0.00	RIDA, TNRC6B, FTO
GO:0030001	GO Biological Processes	metal ion transport	4	11.11	-2.18	0.00	ITPR1, CACNA1I, KCNU1, REP15
GO:0034655	GO Biological Processes	nucleobase-containing compound catabolic process	3	8.33	-2.15	0.00	HK1, RIDA, POP1
GO:0008544	GO Biological Processes	epidermis development	3	8.33	-2.14	0.00	FGFR2, PTHLH, GRHL1
R-HSA-3700989	Reactome Gene Sets	Transcriptional Regulation by TP53	3	8.33	-2.04	0.00	ELL, TNRC6B, SETD9
