# Supplementary material for: Sparse multitask group Lasso for genome-wide association studies
Source: PLoS Comput Biol. 2025 Sep 12;21(9):e1012734. doi: 10.1371/journal.pcbi.1012734 (PMC12448984; doi:10.1371/journal.pcbi.1012734)
Supplement: S10 Table — (PDF) [file pcbi.1012734.s022.pdf]

S10 Table. Summary of enrichment analysis in DisGeNET

GO	Description	#	%	Log10(P)	Log10(q)	Gene Hits
C0425782	Breast size	6	17.00	-14.00	-9.30	ADSL, ESR1, ZNF365, SGSM3, MRTFA, FTO
C2938924	Oestrogen receptor positive breast cancer	9	25.00	-8.20	-4.00	ESR1, FGFR2, MAP3K1, TGFBR2, MRPS30, ZNF365, TOX3, FTO, CCDC170
C3642345	Luminal A Breast Carcinoma	6	17.00	-7.60	-3.50	ESR1, FGFR2, MAP3K1, TGFBR2, TOX3, CCDC170
C2242776	Plexiform leiomyoma	4	11.00	-6.80	-2.90	ITPR1, TNRC6B, MRTFA, NEK10
C4733092	estrogen receptor-negative breast cancer	7	19.00	-6.70	-2.90	ESR1, FGFR2, MAP3K1, PTHLH, TGFBR2, TOX3, CCDC170
C1257931	Mammary Neoplasms, Human	7	19.00	-5.60	-1.90	ESR1, FGFR2, MAP3K1, PTHLH, ZNF365, TOX3, FTO
C4704874	Mammary Carcinoma, Human	7	19.00	-5.50	-1.80	ESR1, FGFR2, MAP3K1, PTHLH, ZNF365, TOX3, FTO
C0042133	Uterine Fibroids	7	19.00	-5.40	-1.80	ESR1, ITPR1, PTHLH, TNRC6B, SGSM3, MRTFA, NEK10
C3642346	Luminal B Breast Carcinoma	4	11.00	-5.20	-1.60	ESR1, FGFR2, MAP3K1, TOX3
C0154084	Stage 0 Breast Carcinoma	3	8.30	-5.10	-1.60	ESR1, FGFR2, TOX3
C0042834	Vital capacity	6	17.00	-5.00	-1.60	ESR1, FGFR2, MAP3K1, PTHLH, ZNF365, TOX3, FTO
C0489786	Height	5	14.00	-5.00	-1.60	ESR1, FGFR2, MAP3K1, PTHLH, TOX3, CCDC170
C0149782	Squamous cell carcinoma of lung	7	19.00	-4.90	-1.50	ESR1, FGFR2, MAP3K1, TOX3
C1691215	Penile hypospadias	4	11.00	-4.80	-1.50	ESR1, FGFR2, MAP3K1, PTHLH, ZNF365, TOX3, FTO
C4520821	Stage 0 Breast Cancer AJCC v6 and v7	3	8.30	-4.80	-1.50	ESR1, FGFR2, MAP3K1, TOX3
C0428883	Diastolic blood pressure	6	17.00	-4.60	-1.30	ESR1, ITPR1, PTHLH, TNRC6B, SGSM3, MRTFA, NEK10
C0152459	Linear atrophy	4	11.00	-4.50	-1.30	ESR1, FGFR2, MAP3K1, PTHLH, ZNF365, TOX3, FTO
C0346163	Endometrioid carcinoma ovary	3	8.30	-4.50	-1.20	ESR1, FGFR2, MAP3K1, PTHLH, ZNF365, TOX3, FTO
C0220620	Gastrointestinal Carcinoid Tumor	5	14.00	-4.40	-1.20	ESR1, FGFR2, MAP3K1, PTHLH, TOX3, CCDC170
C0221357	Brachydactyly	5	14.00	-4.40	-1.20	ESR1, FGFR2, MAP3K1, PTHLH, TOX3, CCDC170
