# Supplementary material for: Sparse multitask group Lasso for genome-wide association studies
Source: PLoS Comput Biol. 2025 Sep 12;21(9):e1012734. doi: 10.1371/journal.pcbi.1012734 (PMC12448984; doi:10.1371/journal.pcbi.1012734)
Supplement: S11 Table — (PDF) [file pcbi.1012734.s023.pdf]

S11 Table. Summary of enrichment analysis in Cell Type Signatures

GO	Description	#	%	Log10(P)	Log10(q)	Gene Hits
M40312	DESCARTES FETAL THYMUS THYMIC EPITHELIAL CELLS	5	14.00	-5.00	-1.60	FGFR2, PAX9, CACNA1I, ASTN2, TOX3
M39241	LAKE ADULT KIDNEY C22 ENDOTHELIAL CELLS GLOMERULAR CAPILLARIES	3	8.30	-3.20	-0.41	EBF1, TGFBR2, PPFIBP1
M39026	FAN EMBRYONIC CTX EX 4 EXCITATORY NEURON	3	8.30	-3.00	-0.31	ITPR1, PPFIBP1, CACNA1I
M39234	LAKE ADULT KIDNEY C15 CONNECTING TUBULE	3	8.30	-3.00	-0.31	ITPR1, PPFIBP1, TOX3
M40237	DESCARTES FETAL LUNG SQUAMOUS EPITHELIAL CELLS	3	8.30	-2.70	-0.16	PAX9, ZNF365, GRHL1
M39233	LAKE ADULT KIDNEY C14 DISTAL CONVOLUTED TUBULE	3	8.30	-2.70	-0.16	ITPR1, TNRC6B, TOX3
M39136	GAO ESOPHAGUS 25W C1 CILIATED EPITHELIAL CELLS	4	11.00	-2.70	-0.15	ASTN2, CCDC170, NEK10, SSBP4
M39222	LAKE ADULT KIDNEY C3 PROXIMAL TUBULE EPITHELIAL CELLS S1 S2	3	8.30	-2.60	-0.12	PPFIBP1, TNRC6B, ASTN2
