# Supplementary material for: Sparse multitask group Lasso for genome-wide association studies
Source: PLoS Comput Biol. 2025 Sep 12;21(9):e1012734. doi: 10.1371/journal.pcbi.1012734 (PMC12448984; doi:10.1371/journal.pcbi.1012734)
Supplement: S12 Table — (PDF) [file pcbi.1012734.s024.pdf]

S12 Table. Summary of enrichment analysis in PaGenBase

GO	Description	#	%	Log10(P)	Log10(q)	Gene Hits
PGB:00073	Tissue-specific: salivary gland	3	8.30	-3.10	-0.35	FGFR2, SGSM3, MIER3
