# Supplementary material for: Sparse multitask group Lasso for genome-wide association studies
Source: PLoS Comput Biol. 2025 Sep 12;21(9):e1012734. doi: 10.1371/journal.pcbi.1012734 (PMC12448984; doi:10.1371/journal.pcbi.1012734)
Supplement: S13 Table — (PDF) [file pcbi.1012734.s025.pdf]

S13 Table. Summary of enrichment analysis in Transcription Factor Targets

GO	Description	#	%	Log10(P)	Log10(q)	Gene Hits
M12934	BRN2 01	4	11.00	-3.70	-0.78	FGFR2, PTLH, ELL, ZMIZ1
M8280	NFAT Q4 01	4	11.00	-3.60	-0.65	EBF1, PTHLH, CCDC91, MIER3
M402	TTCYRGAA UNKNOWN	4	11.00	-3.20	-0.35	ESR1, PTHLH, CACNA1I, GRHL1
M13600	OCT1 07	3	8.30	-3.00	-0.31	FGFR2, CCDC91, NEK10
M3128	AAANWWTGC UNKNOWN	3	8.30	-2.80	-0.21	EBF1, ESR1, FGFR2
M7165	ACTAYRNNNCCCR UNKNOWN	4	11.00	-2.70	-0.14	ITPR1, RIDA, POP1, SGSM3
M10921	AREB6 03	3	8.30	-2.40	0.00	EBF1, MRTFA, SSBP4
M3437	STAT Q6	3	8.30	-2.40	0.00	FGFR2, PTHLH, SSBP4
M15623	SOX5 01	3	8.30	-2.40	0.00	EBF1, FGFR2, ZNF365
M9026	AP4 Q5	3	8.30	-2.40	0.00	ESR1, TGFBR2, ZMIZ1
M5959	IK1 01	3	8.30	-2.30	0.00	ITPR1, CCDC91, MRTFA
M613	TGANNYRGCA TCF11MAFG 01	3	8.30	-2.20	0.00	ESR1, RIDA, POP1
M1328	WTGAAAT UN-KNOWN	4	11.00	-2.20	0.00	PAX9, PTHLH, CCDC91, MIER3
