# Supplementary material for: Sparse multitask group Lasso for genome-wide association studies
Source: PLoS Comput Biol. 2025 Sep 12;21(9):e1012734. doi: 10.1371/journal.pcbi.1012734 (PMC12448984; doi:10.1371/journal.pcbi.1012734)
Supplement: Attachment — Submitted filename: SMuGLasso_ReviewerResponses.pdf [file pcbi.1012734.s027.pdf]

We thank the editor and reviewers for their valuable time, thoughtful comments, and constructive feedback on our manuscript. We appreciate the effort each reviewer has made to evaluate our work and provide suggestions that have helped us improve the clarity of the paper. We have provided a marked document titled "Revised Manuscript with Track Changes.pdf", in which deletions are shown in red and additions in blue. For each reviewer comment, we indicate the corresponding line number and section name in this marked document to help locate the changes.

Answers to reviewer 1

1. The original MuGLasso requires additional post-processing steps to discern task-specific LD groups. What are the post-processing steps? And are they also be used for MuGLasso in the comparison experiments?

Thank you for this important question. In the MuGLasso framework, task-specific LD group selection is not directly built into the optimization process. To identify task-specific LD groups, a simple post-processing step is applied: for each task, we examine the regression coefficients estimated for each LD group and remove those groups whose coefficients are near zero (typically below a small threshold such as 10^{-2} or 10^{-3}). This filters out non-contributory groups for individual tasks. And yes, we applied this same post-processing procedure to MuGLasso in our comparison experiments to ensure a fair evaluation of task-specific group selection across both methods. We have clarified this point in the revised manuscript (Introduction, lines 29–30, Section Materials and methods - General framework, lines 85–87 and Section Results, lines 475 –478) to avoid confusion.

2. In the model, is X used to denote the raw genotypes of SNPs, or the normalized genotypes?

In our model, X denotes the raw genotypes of SNPs, typically encoded as 0, 1, or 2 based on the number of reference alleles. We did not apply normalization to the genotype matrix prior to model fitting. We have clarified this point in the revised manuscript (Section Notations, lines 99–101).

3. How to select hyperparameters lambda 1 and lambda 2?

We used cross-validation on simulated data generated by the GWAS simulator to select the best values for λ_1 and λ_2. The simulator uses real HapMap3 genotypes (YRI and CEU), which share similar LD structure with real datasets such as DRIVE. Due to computational constraints with the real DRIVE data, we applied the same hyperparameters identified from simulation to the real data analysis. For the *Arabidopsis thaliana* dataset, which involves plant genotypes, we performed cross-validation directly on the data (without the stability selection step) to select λ_1 and λ_2. The values obtained were very close to those from simulation-based tuning. We have detailed this in the revised manuscript (Section Experiments - Comparison partners, lines 285–297).

4. How to pre-define the LD groups?

We thank the reviewer for raising this point. LD groups are defined based on

adjacency-constrained hierarchical clustering of SNPs, which groups variants by their local correlation structure. Specifically, we used the adjclust R package to perform this clustering on each chromosome and subpopulation in the simulated and DRIVE datasets. For the *Arabidopsis thaliana* dataset, due to the very large number of SNPs, we first partitioned each chromosome into independent LD blocks using the `snpldsplit` function from the bigsnpr package, and then applied adjclust within each block. After obtaining LD groups per population, we merged the group boundaries across populations to construct a common set of shared LD groups for analysis. This information is currently detailed in the Supporting Information. However, in response to the reviewer's comment, we agree that it is important for readers and have moved a brief summary of this process into the main text (Section Materials and methods - General framework lines 64–71) to improve clarity.

5. Are you using different variable selection methods on SNPs after LD pruning? How is the performance on SNPs without pruning?

LD pruning removes highly correlated SNPs within LD blocks but it does not significantly reduce or increase the number of LD groups, since those SNPs tend to cluster together regardless.

6. Why different r2 thresholds are used for datasets in LD pruning?

We thank the reviewer for this question. The choice of r^2 thresholds for LD pruning was dataset-specific and based on both prior literature and empirical observations. For the *Arabidopsis thaliana* dataset, we followed the recommended parameters from Grimm et al. (*The Plant Cell*, 2017), using an LD cutoff of $r^2 > 0.75$. These settings are widely adopted in *A. thaliana* GWAS. For the simulated data and the DRIVE dataset, we used a more stringent cutoff of $r^2 > 0.85$. This threshold provided a good trade-off: it retained a large number of SNPs while reducing the genomic inflation factor, especially in the baseline methods (adjusted GWAS, adjusted Lasso, and adjusted group Lasso). This helped control for population stratification and allowed for a fairer comparison with MuGLasso and SMuGLasso.

7. In Figure 2, do runtimes of SMuGLasso and MuGLasso also incorporate the stability selection steps?

Yes, the runtimes reported in Figure 2 for both SMuGLasso and MuGLasso do include the stability selection steps. Thanks to the use of screening rules—in particular, the gap safe rules for group Lasso we were able to make stability selection computationally feasible. We add a brief note about this point in the manuscript (Section Results lines 369-370).

Minor issue: Page 12, Line 363: For the *Arabidopsis thaliana* dataset (see ??)

Thank you for pointing this out. The citation has been corrected in the revised manuscript.

Answers to reviewer 2

1. The definition of $B^{(g)}$ in the penalty term is not clearly stated. It would be helpful to explain that $B^{(g)} \in \mathbb{R}^{p_g \times T}$ refers to the "submatrix" of the coefficient matrix B corresponding to SNPs in LD group g, across all tasks. This clarification would improve the readability of the optimization formulation.

We thank the reviewer for this helpful suggestion. We agree that clarifying the definition of $B^{(g)}$ improves the readability of the optimization formulation. We have now explicitly stated that $B^{(g)} \in \mathbb{R}^{p_g \times T}$ refers to the *submatrix* of the coefficient matrix $B \in \mathbb{R}^{p \times T}$ corresponding to the SNPs in LD group g, across all tasks. This clarification has been added in Section Materials and methods - MuGLasso and its formulation (lines 111-112) where the penalty terms are introduced.

2. There is some potential ambiguity in the notation of regression coefficients. The manuscript uses both $\beta_j^{(t)}$ and a matrix $B \in \mathbb{R}^{p \times T}$, but does not explicitly state how they relate. For example, can we say $B_{jt} = \beta_j^{(t)}$. A brief clarification of this indexing convention would help avoid confusion.

We thank the reviewer for pointing this out. To clarify the relationship between notations, we now explicitly state that the matrix $B \in \mathbb{R}^{p \times T}$ contains all regression coefficients, where each entry B_{jt} corresponds to the coefficient $\beta_j^{(t)}$—that is, the effect of SNP j in task (or population) t. This indexing convention has been clarified in the revised manuscript (Section MuGLasso and its formulation, line 105) to avoid ambiguity.

3. It is also unclear whether the LD groups are assumed to be non-overlapping and exhaustive. If each SNP is assigned to exactly one group, it would be beneficial to state this assumption explicitly.

We thank the reviewer for this observation. Yes, in our framework, LD groups are non-overlapping and exhaustive — each SNP is assigned to exactly one LD group, and no SNP belongs to more than one group. We have now made this assumption explicit in the manuscript (Section Materials and methods - General framework lines 59–66) to avoid confusion.

4. Finally, the manuscript transitions rather abruptly from the matrix-based formulation to the vectorized reformulation using $\tilde{X}$, $\tilde{y}$, and $\boldsymbol{b} \in \mathbb{R}^{pT}$. Providing a clearer explanation of the purpose and advantages of this reformulation, such as improved computational efficiency or compatibility with standard optimization algorithms, would enhance the clarity of the presentation.

We thank the reviewer for this important observation. Our main reason for adopting this reformulation is to express the multi-input, single-output formulation of our problem in a standard optimization framework. Classical multitask Lasso settings typically assume multiple outputs (e.g., multiple phenotypes) observed on the same input samples. In contrast, our setting involves multiple subpopulations (tasks) that are measured on a shared output phenotype. To represent this structure consistently, we stack the SNP matrices from different populations into a block-diagonal matrix $\tilde{X}$ and concatenate the phenotype vectors into a single vector $\tilde{y}$. This yields a single-task regression model where

each block of features corresponds to a different population. This reformulation captures the independence between populations and also makes our optimization problem compatible with standard solvers that support group-lasso and sparse-group-lasso penalties. We have clarified this motivation in the revised manuscript to improve the clarity of Section Materials and methods - MuGLasso and its formulation Page 4 - After line 112 to the end of the page.

5. The SMuGLasso formulation introduces an additional ℓ_1 penalty, but the motivation for this extension is only briefly mentioned. It would be helpful to elaborate further on the practical and theoretical benefits of adding this term, such as improving sparsity at the group level or reducing false positives.

Thank you for the helpful comment. We added the ℓ_1 penalty in SMuGLasso to improve the selection of LD groups that are specific to each population. In MuGLasso, the group penalty is applied in the same way for all populations, so it cannot easily tell which LD group is relevant to which population. To do that, MuGLasso needs a post-processing step after training to remove small coefficients. With the extra ℓ_1 penalty, SMuGLasso can turn off an LD group in some populations while keeping it active in others. This makes the model better at selecting genetic variants that are specific to each population. It also avoids the need for post-processing. We now explain this motivation more clearly in the Methods Section of the revised paper (Section General framework lines 75–77 and Section SMuGLasso lines 118–119). In practice, this dual-penalty formulation leads to a more precise and interpretable identification of associated SNPs, and maintains as well computational feasability through the use of coordinate descent and gap safe screening rules.

6. The optimization problem is written with nested summations, but some indexing appears inconsistent or difficult to follow. For example, the summation over $t = 1, \dots, T$ and $m = 1, \dots, n_t$ lacks clear delimiters and formatting, which makes the structure of the loss term harder to interpret. Please clarifying this for improved readability.

We thank the reviewer for pointing out the formatting issue in the loss function. In the revised manuscript, we have reformatted the expression (Section MuGLasso and its formulation, Equation (1) and Section SMuGLasso, Equation (2)) to clarify the summation domains and indexing for better readability.

7. The penalty terms are written as $\lambda_1 \sum_{g=1}^{G} \sqrt{p_g} \|B^{(g)}\|_F + \lambda_2 \sum_{g=1}^{G} \sqrt{p_g} \|B^{(g)}\|_1$, which raises a question about how $\|B^{(g)}\|_1$is defined when $B^((g))$ is a matrix. If it denotes the elementwise ℓ_1 norm, this should be explicitly stated. Also, some justification or discussion of the combined Frobenius and ℓ_1 penalties in the context of interpretability or model behavior would be beneficial.

We thank the reviewer for this important observation. We confirm that the term $\|B^{(g)}\|_1$ refers to the *elementwise* ℓ_1 norm of the matrix $B^{(g)}$, that is:$\|B^{(g)}\|_1 = \sum_{j=1}^{p_g} \sum_{t=1}^{T} \left| B_{jt}^{(g)} \right|$. We have clarified this explicitly in the revised manuscript (Section SMuGLasso - Problem formulation, lines 121–122) to remove any ambiguity. Regarding the combination of the Frobenius norm and the elementwise ℓ_1 norm, this dual-penalty structure allows SMuGLasso to capture both group-level and within-group sparsity. The Frobenius norm $\|B^{(g)}\|_F$

promotes selection of entire LD-groups across tasks, while the elementwise ℓ_1 norm $\|B^{(g)}\|_1$ allows finer control by zeroing out coefficients at the population level. This leads to more interpretable models by identifying both shared and population-specific effects within LD-groups. It also mitigates false positives by avoiding the inclusion of full groups when only a subset of coefficients are truly informative. We have added a short explanation of this behavior in the SMuGLasso - Problem formulation Section (lines 122–139).

8. A reference to Appendix S1 is given for implementation details, but it would be helpful to include a brief high-level summary in the main text for readers who want an overview without immediately turning to the appendix.

We thank the reviewer for this suggestion. We agree that providing a brief summary of the implementation details in the main text improves readability and the understanding of the method. In the revised manuscript, we have added an overview of the main steps of the SMuGLasso implementation—covering population assignment, LD-group formation, model fitting with the dual penalty, and stability selection (We add a subsection General framework in the Materials and methods Section in line 55), while keeping the more technical details in Appendix S1.

9. The manuscript offers a comprehensive comparison of SMuGLasso with a variety of baseline and benchmark methods. However, the presentation of the comparison strategies could benefit from greater clarity and structure. For example, the distinctions among the "Adjusted," "Stratified," and "Group" variants of Lasso and Group Lasso are introduced in a dense block of text that can be difficult to follow. Consider summarizing these comparison partners in a table or a brief schematic, which would help readers quickly grasp the methodological differences.

We agree that the description of baseline and comparison methods was dense and may be difficult to follow at a glance. In the revised manuscript, we have added a summary table Table 4 - Page 10 that categorizes each method by how it handles population stratification, includes or not LD grouping, and adjust or not for the phenotype. This table helps clarify the key methodological differences between SMuGLasso, MuGLasso, and the Lasso-based and Group Lasso-based baselines as suggested by the reviewer.

10. The procedure for generating PCA-adjusted phenotypes, particularly the step of subtracting the fitted values from the observed binary outcomes in logistic regression, is unconventional. It would be helpful to justify this approach or cite prior work where it has been validated or effectively applied, especially in the context of binary phenotypes.

We thank the reviewer for raising this point. We agree that generating PCA-adjusted phenotypes rather than including PCs as covariates can be surprising in the context of a case-control study. However, this has been done before (see e.g. Abegaz et al. (2021)) and we do not see any other way to correct for population structure in an ℓ-1 based regularization (either Lasso or Group Lasso). Indeed, using the PCs as covariates does not guarantee that they will be selected, and confounded SNPs might end up being selected instead. In addition, this adjustment method effectively reduced the effect of population stratifica-

tion on our data, achieving an inflation factor close to 1. We have clarified this methodological choice in the revised manuscript in Section Experiments - Comparison partners, lines 278–280

11. The manuscript notes the limitations of linear mixed models (e.g., FastLMM) for feature selection, which is a relevant point. However, this discussion could be strengthened by clarifying whether any efforts were made to compare performance against mixed models in terms of predictive accuracy or robustness, even if feature selection per se was not directly compatible.

We thank the reviewer for this thoughtful comment. We agree that linear mixed models (LMMs), such as FastLMM, are highly relevant in GWAS for handling population structure and polygenic background. In our work, we focus on multivariate models based on sparse regression approaches that directly yield sets of selected features, rather than association statistics as produced by methods like FastLMM. However, we have now included FastLMM in our comparative analyses. To enable this comparison, we computed the stability index for FastLMM by considering SNPs consistently detected as significant across resampled datasets. Additionally, we now compute the stability index for traditional GWAS results in the same way as for FastLMM. We observe indeed the strengths of FastLMM and classical GWAS in terms of stability and robustness, while also highlighting two key limitations: first, their inability to identify population-specific signals, and second, the fact that they detect only a small number of SNPs due to the lack of statistical power caused by the curse of dimensionality in GWAS data. These points are clarified in the revised manuscript Section Experiments - Comparison partners, lines 254–258, Section Results (lines 426–428, 432–434 and 440–454), Table 5, Table 6, Fig 1 and Fig 2.

12. It is good to see that cross-validation is used consistently across methods, but it would be helpful to mention how the folds were selected (e.g., stratified by population or phenotype) and whether the same splits were used across all methods for a fair comparison.

In our experiments, we ensured indeed consistent cross-validation across all methods. For methods that jointly handle all subpopulations (SMuGLasso, MuGLasso, Adjusted Lasso/Group Lasso), we used 3-fold cross-validation with stratification based on a composite of both population and phenotype label. This ensured that each fold preserved the joint distribution of population structure and phenotype classes, in order to prevent imbalances through folds, such as folds dominated by a single population or phenotype. For methods applied separately within each population (Stratified Lasso/ Group Lasso), cross-validation was performed independently within each subpopulation. In those cases, we used stratification based only on phenotype labels, as population matter is already fixed. We have clarified this cross-validation procedure in the manuscript (Section Experiments - Comparison partners, lines 285–297) to clarify the evaluation setup.

13. The measure of feature selection stability is interesting and valuable, but it would benefit from further elaboration. For instance, is Pearson correlation among binary indicator vectors the most appropriate metric for stability? Are

there alternative or complementary measures (e.g., Jaccard similarity) that were considered?

We thank the reviewer for this question. Based on the work of Nogueira and Brown (2015), we compared several stability indices and focused on those that satisfy key properties like boundedness, consistency, and correction for chance. Among these, the Pearson-based index and the variance-based index from Nogueira et al. (2018) performed best. We chose the Pearson-based index because it accounts for the exact number of selected features in each run, which makes it more accurate when selection sizes vary. By contrast, the Jaccard indexdoes not correct for random overlap, which is important in high-dimensional settings. We have added a short explanation in the paper (Section Experiments - Comparison partners, lines 303–306) to clarify this choice.

14. The description of FUMA and Metascape usage is generally clear. However, it would be helpful to briefly explain the biological rationale for choosing specific parameters, such as the 10 kb mapping window and the p-value threshold of 5×10^8, especially for readers who may be less familiar with standard GWAS annotation practices.

We thank the reviewer for this suggestion. The 10 kb mapping window and the genome-wide significance threshold of 5×10^{-8} were chosen based on common practice in GWAS annotation studies. The 10 kb window is widely used in tools like FUMA to capture nearby genes affected by associated SNPs, while remaining conservative to focus on top candidate genes and minimizing potential false positives. The 5×10^{-8} threshold is a standard cutoff to identify high-confidence associations in GWAS to control for multiple-testing. In our FUMA analysis, we assigned the selected SNPs artificial p-values below this threshold of 5×10^{-10} to ensure they are processed as significant and mapped to genes. We have clarified this in the manuscript (Section Experiments - Biological interpretation, lines 313–317).

15. While Metascape is a popular tool, some brief comment on why it was chosen over alternatives (e.g., DAVID, GSEA, Enrichr) might help contextualize the analysis choices.

We chose Metascape because it offers an integrated, up-to-date platform combining multiple annotation databases, along with multiple visual outputs. It supports multiple species, including *Arabidopsis thaliana*. We have added a short comment about this in the updated manuscript (Section Experiments - Biological interpretation, lines 331–334).

16. Since *Arabidopsis thaliana* is included, it may be worth clarifying whether any plant-specific databases or annotations were used beyond specifying the species in Metascape, especially as many enrichment tools are human-centric by default.

In our study, we selected *Arabidopsis thaliana* as the reference species in Metascape, which supports functional enrichment and PPI analysis for this plant specie. We did not use additional plant-specific databases, as Metascape provided sufficient annotations for our gene lists. We have added a note in the manuscript (Section Experiments - Biological interpretation, lines 333–334) to clarify this.

17. The description of the protein–protein interaction (PPI) network analysis could benefit from further clarification regarding its biological relevance. It might be helpful to briefly explain why constructing a PPI network is meaningful within the context of this study. Since this is a methodology paper, I found myself a bit confused at first. This should be to add a real data application layer to validate biological findings, correct? Please add a description to differentiate the role of the PPI network in your analysis.

Thank you for the comment. We added the PPI network analysis to give biological meaning to our results. The idea is to check if the genes selected by SMuGLasso (or MuGLasso) are not just statistically significant, but also functionally related. If they are part of the same network, this supports that they may be involved in the same biological process. This gives more confidence that the selected genes are relevant to the disease or trait. We have added a short explanation about this in the manuscript (Section Experiments - Biological interpretation, lines 346–349).

18. The comparative analysis of stability across methods is clear and informative. To enhance the interpretation, it would be helpful to briefly discuss why MuGLasso consistently shows higher stability than SMuGLasso, even though SMuGLasso includes an additional penalty. This could help clarify the trade-off between model sparsity and selection consistency. Also, since the results include both LD group level and single SNP level selection, a short explanation of how these selection levels might influence the stability index would improve the comparison. For example, do LD group-based methods naturally produce more stable results due to grouping?

MuGLasso shows higher stability than SMuGLasso because it selects more features overall and does not include the extra ℓ_1 penalty. The additional penalty in SMuGLasso makes the model sparser, which improves precision but can slightly reduce consistency across subsamples. This is a known trade-off in sparse models. Indeed, it is how Zou & Hastie motivate their proposition of the elastic net in their 2005 paper *Regularization and variable selection via the elastic net* (see also Hastie, Tibshirani & Wainwright (2015), *Statistical Learning with Sparsity*, chapter 4). Also, methods based on LD-group selection are generally more stable than those working at the single SNP level. Grouping SNPs reduces the number of possible selection choices, which leads to more consistent selections across subsamples. We clarified both points in the manuscript (Section Results, lines 456–464).

19. Results section would benefit from clearer organization. While there are subsections, the titles could help differentiate the simulation results from the real data analysis for biological validation (GSEA and PPI). Adding specific titles for each subsection would make it much easier for readers to follow the flow of the analysis and clearly distinguish between the different parts.

We thank the reviewer for the helpful suggestion. We have revised the Results section to include clearer and more specific subsection titles to better separate the simulation results from the real data analysis and biological validation steps.

20. How many simulation replicates were used in your experiment? I may

have missed this, but I couldn't find the information in the manuscript. Additionally, in the simulation settings, could you clarify how the null and non-null hypotheses or scenarios were defined? These should be explicitly stated, along with the criteria used to determine true positives and false positives.

We thank the reviewer for this important question. We used a single simulation replicate in our study. Due to the large dataset size (4 000 samples and 50 000 SNPs) and the computational cost of running all comparison methods with stability selection, generating multiple independent simulation replicates was not feasible within a reasonable time frame. Regarding the simulation design: we predefined 200 causal SNPs (non-null hypotheses): 50 specific to CEU, 50 to YRI, and 100 shared across both populations. These were randomly located on chromosomes 12, 19, 21, and 22. The null hypotheses correspond to all the remaining SNPs. We considered a SNP (or LD-group) to be a true positive if it overlapped with the predefined causal SNPs (or the LD-groups containing them). Any selected SNP or LD-group outside this predefined causal set was counted as a false positive. We have clarified this in the manuscript (Section Experiments - Data - Simulated data, lines 195–198 and lines 203–208).
