# Supplementary material for: Sparse multitask group Lasso for genome-wide association studies
Source: PLoS Comput Biol. 2025 Sep 12;21(9):e1012734. doi: 10.1371/journal.pcbi.1012734 (PMC12448984; doi:10.1371/journal.pcbi.1012734)
Supplement: Attachment — Submitted filename: PLOS_CB_ReviewerComments.docx [file pcbi.1012734.s026.docx]

1. The definition of $B^{(g)}$ in the penalty term is not clearly stated. It would be helpful to explain that $B^{(g)}$ ∈ $\mathbb{R}^{P_{g}\times T}$ refers to the “submatrix” of the coefficient matrix $B$ corresponding to SNPs in LD group $g$, across all tasks. This clarification would improve the readability of the optimization formulation.

2. There is some potential ambiguity in the notation of regression coefficients. The manuscript uses both $\beta_{j}^{(t)}$ and a matrix $B$ ∈ $\mathbb{R}^{p\times T}$, but does not explicitly state how they relate. For example, can we say $B_{jt}=\beta_{j}^{(t)}$. A brief clarification of this indexing convention would help avoid confusion.

3. It is also unclear whether the LD groups are assumed to be non-overlapping and exhaustive. If each SNP is assigned to exactly one group, it would be beneficial to state this assumption explicitly.

4. Finally, the manuscript transitions rather abruptly from the matrix-based formulation to the vectorized reformulation using $\tilde{X}$, $\tilde{y},$ and $b$ ∈ $\mathbb{R}^{pT}$. Providing a clearer explanation of the purpose and advantages of this reformulation, such as improved computational efficiency or compatibility with standard optimization algorithms, would enhance the clarity of the presentation.

5. The SMuGLasso formulation introduces an additional $\mathcal{l}_{1}$ penalty, but the motivation for this extension is only briefly mentioned. It would be helpful to elaborate further on the practical and theoretical benefits of adding this term, such as improving sparsity at the group level or reducing false positives.

6. The optimization problem is written with nested summations, but some indexing appears inconsistent or difficult to follow. For example, the summation over $t=1,\ldots,T$ and $m=1,\ldots,n_{t}$ lacks clear delimiters and formatting, which makes the structure of the loss term harder to interpret. Please clarifying this for improved readability.

7. The penalty terms are written as $\lambda_{1}\sum_{g=1}^{G} \sqrt{p_{g}}\left\| B^{(g)} \right\|_{F}+\lambda_{2}\sum_{g=1}^{G} \sqrt{p_{g}}\left\| B^{(g)} \right\|_{1}$, which raises a question about how $\left\| B^{(g)} \right\|_{1}$is defined when $B^{(g)}$is a matrix. If it denotes the elementwise $\mathcal{l}_{1}$ norm, this should be explicitly stated. Also, some justification or discussion of the combined Frobenius and $\mathcal{l}_{1}$ penalties in the context of interpretability or model behavior would be beneficial.

8. A reference to Appendix S1 is given for implementation details, but it would be helpful to include a brief high-level summary in the main text for readers who want an overview without immediately turning to the appendix.

9. The manuscript offers a comprehensive comparison of SMuGLasso with a variety of baseline and benchmark methods. However, the presentation of the comparison strategies could benefit from greater clarity and structure. For example, the distinctions among the “Adjusted,” “Stratified,” and “Group” variants of Lasso and Group Lasso are introduced in a dense block of text that can be difficult to follow. Consider summarizing these comparison partners in a table or a brief schematic, which would help readers quickly grasp the methodological differences.

10. The procedure for generating PCA-adjusted phenotypes, particularly the step of subtracting the fitted values from the observed binary outcomes in logistic regression, is unconventional. It would be helpful to justify this approach or cite prior work where it has been validated or effectively applied, especially in the context of binary phenotypes.

11. The manuscript notes the limitations of linear mixed models (e.g., FastLMM) for feature selection, which is a relevant point. However, this discussion could be strengthened by clarifying whether any efforts were made to compare performance against mixed models in terms of predictive accuracy or robustness, even if feature selection per se was not directly compatible.

12. It is good to see that cross-validation is used consistently across methods, but it would be helpful to mention how the folds were selected (e.g., stratified by population or phenotype) and whether the same splits were used across all methods for fair comparison.

13. The measure of feature selection stability is interesting and valuable, but it would benefit from further elaboration. For instance, is Pearson correlation among binary indicator vectors the most appropriate metric for stability? Are there alternative or complementary measures (e.g., Jaccard similarity) that were considered?

14. The description of FUMA and Metascape usage is generally clear. However, it would be helpful to briefly explain the biological rationale for choosing specific parameters, such as the 10 kb mapping window and the p-value threshold of 5×10⁻⁸, especially for readers who may be less familiar with standard GWAS annotation practices.

15. While Metascape is a popular tool, some brief comment on why it was chosen over alternatives (e.g., DAVID, GSEA, Enrichr) might help contextualize the analysis choices.

16. Since Arabidopsis thaliana is included, it may be worth clarifying whether any plant-specific databases or annotations were used beyond specifying the species in Metascape, especially as many enrichment tools are human-centric by default.

17. The description of the protein–protein interaction (PPI) network analysis could benefit from further clarification regarding its biological relevance. It might be helpful to briefly explain why constructing a PPI network is meaningful within the context of this study. Since this is a methodology paper, I found myself a bit confused at first. This should be to add a real data application layer to validate biological findings, correct? Please add a description to differentiate the role of the PPI network in your analysis.

18. The comparative analysis of stability across methods is clear and informative. To enhance the interpretation, it would be helpful to briefly discuss why MuGLasso consistently shows higher stability than SMuGLasso, even though SMuGLasso includes an additional penalty. This could help clarify the trade-off between model sparsity and selection consistency. Also, since the results include both LD group level and single SNP level selection, a short explanation of how these selection levels might influence the stability index would improve the comparison. For example, do LD group-based methods naturally produce more stable results due to grouping?

19. Results section would benefit from clearer organization. While there are subsections, the titles could help differentiate the simulation results from the real data analysis for biological validation (GSEA and PPI). Adding specific titles for each subsection would make it much easier for readers to follow the flow of the analysis and clearly distinguish between the different parts.

20. How many simulation replicates were used in your experiment? I may have missed this, but I couldn't find the information in the manuscript. Additionally, in the simulation settings, could you clarify how the null and non-null hypotheses or scenarios were defined? These should be explicitly stated, along with the criteria used to determine true positives and false positives.
